# Native Endophytes of *Tripterygium wilfordii*-Mediated Biotransformation Reduces Toxicity of Celastrol

**DOI:** 10.3389/fmicb.2022.810565

**Published:** 2022-05-25

**Authors:** Ping-yang Ma, Wei-ling Geng, Hong-yan Ji, Bang-wen Yue, Cheng Liu, Sa Wang, Zhi-bo Jiang, Jing Chen, Xiu-li Wu

**Affiliations:** ^1^College of Pharmacy, Ningxia Medical University, Yinchuan, China; ^2^Department of Pharmaceutics, General Hospital of Ningxia Medical University, Yinchuan, China; ^3^Key Laboratory for Chemical Engineering and Technology, School of Chemistry and Chemical Engineering, State Ethnic Affairs Commission, North Minzu University, Yinchuan, China; ^4^Institute of Translational Medicine, Medical College, Yangzhou University, Yangzhou, China

**Keywords:** celastrol, bio-transformation, endophyte, reduce toxicity, hydroxylation

## Abstract

Celastrol (**1**), obtained from the roots of *Tripterygium wilfordii* Hook F., is most likely to become an antitumor drug, but with severe cytotoxicity. Due to the lack of modifiable sites in the structure of celastrol, the structural diversity of the modified products obtained by synthesis in the previous studies is insufficient, which hinders the pace of its patent medicine. This study describes a method of microbial transformation to increase the modification site of celastrol and reduce its toxicity. The screening of endophytes from native plants was introduced in this context, which led to two novel stereoselective oxidation products such as *S*-16-hydroxyl celastrol (**2**) and A-ring aromatized *S*-16-hydroxyl celastrol (**3**), along with a rare 7,9-octadecadienoic acid ester of celastrol (**4**). Their structures were determined by extensive spectroscopic data analysis, especially 1D and 2D NMR. Compared with **1**, compounds **3** and **4** exhibited similar antitumor activity in U251, A549, KG-1, and B16 cell lines. Compound **2** had slightly decreased antitumor activity when compared with compound **1**. Furthermore, compound **2**–**4** showed lower cytotoxicity against BV-2 (about 21-fold lower, **2**: 92.82 μM, **3**: 34.25 μM, and **4**: 74.75 μM vs. celastrol: 4.35 μM), and also identical trends against H9c2 and PC12 cell lines.

## Introduction

Celastrol (**1**) is a quinone methide pentacyclic triterpenoid isolated from the roots of *Tripterygium wilfordii* Hook F., which exhibits multiple promising biological activities, including anticancer, anti-inflammation, anti-obesity, and anti-diabetic activities (Liu et al., 2015; Chen et al., 2018; You et al., 2021). However, it also associated with limitations such as poor water stability (Qi et al., 2014), low bioavailability (Zhang et al., 2012; Shi et al., 2020), narrow therapeutic window, and undesired side effects. These limitations have greatly hindered its clinical application, and have thus attracted considerable interests from pharmacologists and chemists.

In the past two decades, many chemical synthesizers have tried to modify the structure of celastrol through chemical modification, resulting in more than 190 new entities (Sun et al., 2010; He et al., 2020; Hou et al., 2020). The modifiable sites in the structure, however, are very limited, with the most frequent transformations in the structure–activity relationship studies involving the C=O-2, C(OH)-3, CH-6, and COOH-30 groups (Tang et al., 2015; Jiang et al., 2016; Li et al., 2019). Previously, the C-30 carboxylic group was the prime modification site of celastrol. The classical aim of C-30 carboxylic group modification was to improve the water solubility by conjugation with alcohol, amine, amino acid, urea, or carbamate (Shang et al., 2021). Celastrol has also been conjugated with other anticancer agents with different mechanisms of action through the C-30 carboxylic group to generate hybrids with improved activities and reduced toxicity. The derivatization of C-3 hydroxyl group with hydrophilic groups such as piperazine, and C-6 sulfonation and sulfidation could increase antiproliferative activities. Although these derivatives improved the solubility and absorbance, the consequent infertility, cardiotoxicity, and hematopoietic system toxicity should not be ignored. Therefore, it is important to explore the diversity of celastrol derivatives providing alternative sites for chemical modification.

**FIGURE 1:**
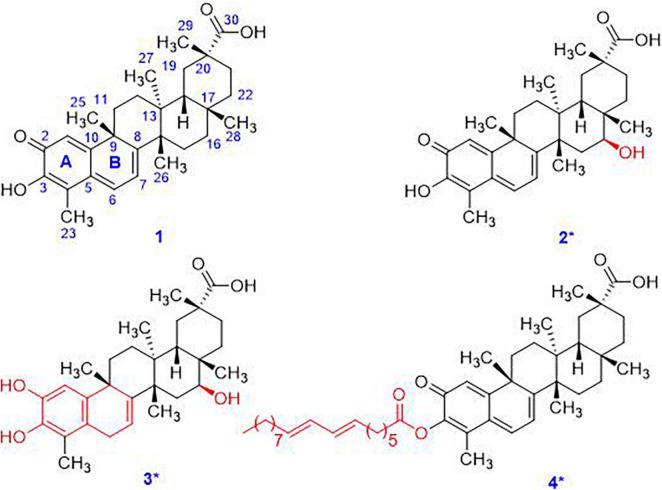
The chemical structures of compounds **1**–**4**. Compounds **2**–**4** were new structures.

Biotransformation and biodegradation of toxic substances has always been one of the self-protection behaviors of organisms (Li and Zheng, 2020). Aldolization, oxidation, and hydroxylation, and some decomposition reactions are the main types of biodegradation mechanisms (Li et al., 2020). However, as celastrol has strong antibacterial properties, common culturable strains in the laboratory could not tolerate it. This study aims to search for celastrol-tolerant symbiotic microorganisms in *T. wilfordii*, the native plant of celastrol. We identified five strains (LGT-1–LGT-5) resistant to celastrol in concentrations of 25–50 mg/150 ml. Among them, LGT-5 could transform celastrol into compounds **2** (Wu et al., 2020) and **3** with novel *S*-16-OH, and compound **4** bearing rare 7,9-octadecadienoic acid ester (Figure 1). Herein, the structural determination of compounds **2**–**4** is described in detail.

## Materials and Methods

### General Experimental Procedures

Nuclear Magnetic Resonance (NMR) spectra were detected at 400 MHz for ^1^H and 100 MHz for ^13^C on Bruker AVIII 400 MHz spectrometers (Bruker Daltonic Inc., Billerica, MA, United States) in methanol-*d*_4_ (CD_3_OD) and chloroform-d (CDCl_3_) with solvent peaks used as references. (–)-HRESIMS data were measured using an Agilent 1290 Infinity II Accurate Mass Q-TOF-LC/MS spectrometer (Agilent Technologies, Santa Clara, CA, United States). Column chromatography (CC) was performed with silica gel (200–300 mesh, Qingdao Marine Chemical Inc., Qingdao, China) and Sephadex LH-20 (Amersham Biosciences Inc., Shanghai, China). Preparative high-performance liquid chromatography (HPLC) was performed by using a Harbor NP7005C pump system (Harbor Sci. & Tech., China) equipped with Mgres/headra C18 (250 × 20 mm, 10 μm, Harbor Sci. &Tech., China). Thin layer chromatography (TLC) was carried out on precoated silica gel GF_254_ glass plates. Spots were visualized under ultraviolet (UV) light or by spraying with 10% H_2_SO_4_ in 95% ethanol (EtOH) followed by heating.

### The Preparation of Celastrol

The root and stem of *T. wilfordii* (10 kg) was crushed into granules of about 3 × 4 mm, and was then extracted three times with 80% EtOH (1 h, v/v, 1:80) using ultrasonic extraction. The 80% residue was suspended in water (H_2_O, 1 L) and then partitioned with ethyl acetate (EtOAc, 3 × 1 L). The EtOAc extract was evaporated under reduced pressure to yield 220.8 g of residue, which was subjected to silica gel CC. Elution was carried out with a petroleum-acetone (Me_2_CO) gradient (100:0–0:100) to produce a crude celastrol fraction (petroleum-Me_2_CO, 100:7) on the basis of TLC analysis. The crude celastrol fraction was further subjected to additional chromatography on Sephadex LH-20 [methanol (MeOH)-dichloromethane (CH_2_Cl_2_), 1:1] to yield celastrol (12.4 g).

### Isolation and Identification of LGT-5

#### Fungus and Cultural Conditions

The fungus LGT-5 was isolated on potato dextrose agar media from fresh *T. wilfordii* collected from Yao County (Dali, Yunnan Province) using a previously described explant culture method and repeated streaking (Wang et al., 2020). This fungus was stored in slants of modified Martin Medium (MMM) (tryptone 5.0 g, yeast extract powder 2.0 g, glucose 20.0 g, K_2_HPO_4_ 1.0 g, MgSO_4_ 0.5 g, agar 20.0 g, distilled water 1 L, pH 6.2–6.5) at 4°C at the Ningxia Medical University, China.

#### Colony Morphology Observation

The endophytic fungus strain LGT-5 from *T. wilfordii* was inoculated in MMM and oat medium (OMA) (oat 30.0 g, agar 20.0 g, distilled water 1 L), respectively. The endophytic fungus was cultured at 28°C for 5–7 days. The colony diameter was recorded and photographed.

Colony morphology was observed by scanning electron microscope. LGT-5 was inoculated in MMM, and a sterile cover glass was inserted into the colony growth medium at an angle of 45°. After 2 days of culture, the cover glass was gently pulled out and washed twice with 0.1 M sodium dimethyl arsenate buffer, and then fixed in 2% glutaraldehyde solution for 2 h (the side with hyphae facing upward). After fixation, the cover glass was washed three times (once every 2 h) in 0.1 M sodium dimethyl arsenate buffer, and finally fixed in 0.1 M sodium dimethyl arsenate buffer at 4°C for more than 12 h. It was then dehydrated with increasing concentrations of ethanol, 30, 50, 70, 80, 90, and 100% ethanol, for 10–15 min for each concentration. The samples were incubated twice with 95% tert-butyl alcohol solution for 15 min each time, and then incubated with 100% tert-butyl alcohol solution for 15 min. Thereafter, the samples were placed in the refrigerator at –20°C for 20 min. After freeze-drying and ion sputtering, the prepared samples were placed under a scanning electron microscope and observed under 10 kV.

#### ITS-18S Sequencing

The universal primers ITS1 and ITS4 were used for polymerase chain reaction (PCR) amplification on PCR amplification system (25 μl): 10 × Taq buffer 2.5 μl, dNTPs (2.5 mmol/l) 2.0 μl, Taq DNA polymerase (Tiangen Biotech Co., Ltd., Beijing, China) 0.2 μl, upstream and downstream primers (10 μmol/l) 1.0 μl, DNA template 2.0 μl, and distilled water supplement to 25 μl. Amplifications were performed using MiniCyclerTM PCR (PERKINEI, PTC-150, MJ Research) with an initial pre-denaturation at 94°C for 3 min; denaturation at 94°C for 30 s, annealing at 56°C for 30 s, extension for 90 s at 72°C for 40 cycles, and finally extension for 5 min at 72°C and preservation at 16°C. The PCR products were purified, recovered and sequenced.

### Biotransformation Process and Isolation of Compounds 2–4

The cultivation of the strains was carried out on a shaker at 28°C and 180 rpm for 2 days in a 250 ml conical bottle containing 150 ml liquid medium (MMM without agar, 40 bottles). Thereafter, 50 mg celastrol was added per bottle and further cultivated for another 5 days. The co-culture supernatant was filtered and partitioned 4 times with EtOAc (v/v, 1:1) and n-BuOH (v/v, 1:1). The EtOAc extract was evaporated under reduced pressure to yield 0.89 g. The EtOAc residue was subjected to silica gel CC eluting with a petroleum-Me_2_CO gradient (100:0–0:100) to produce seven fractions (A-G) on the basis of TLC analysis. Fraction B was fractionated through reversed-phase preparative HPLC using a mobile phase of MeOH-H_2_O (93:7) to yield five fractions (B1–B5) and compound **4** (25.5 mg). Fraction B3 was further separated by preparative RP-HPLC by using MeOH-H_2_O (85: 15 and 82:18) to yield compound **2** (8.9 mg) and B3-2. CC of B3-2 over Sephadex LH-20 [petrol-CHCl_3_-MeOH (5:5:1)] afforded compound **3** (14.6 mg).

### Cytotoxicity Evaluation

Cell counting kit-8 (CCK-8) method was used to screen the antitumor activities on glioma cells U251 (Cheng et al., 2021), lung cancer cell line A549 (Liu et al., 2021), acute myeloid leukemia cells KG-1 (Naimi et al., 2019), and mouse melanoma cells B16 (de Souza et al., 2021). The cytotoxicity was evaluated using the PC12 (Hu et al., 2016), BV-2 (Mi et al., 2019), and H9c2 (Hu et al., 2016) cell lines using CCK-8 assay.

#### Test Sample Preparation

Compounds **1**–**4** were prepared with dimethyl sulfoxide (DMSO, solvent) to obtain 50 mmol/l mother liquor, and then diluted with complete medium to obtain concentrations of 8.0, 4.0, 2.0, or 1.0 μmol/l. The complete medium consisted of 90% DMEM basic medium supplemented with 10% fetal bovine serum.

#### Culture Conditions

A 100 μl test cell suspension (containing 5 × 10^3^ cells) was added to each well of a 96-well plate and incubated at 37°C, 5% CO_2_ for 24 h. The supernatant was then discarded, 100 μl sample solution was added, and the cells were incubated for 48 h. Thereafter, the supernatant was discarded, and 3 holes set for each sample solution. The samples were washed with PBS buffer, stained with CCK-8 (2 h), and enzyme-linked immunosorbent assay used to determine the absorbance at 490 nm.

Cell proliferation inhibition rate (%) = (OD value administration hole OD value control hole)/OD value blank hole × 100%.

Cell survival rate (%) = (1 – cell proliferation inhibition rate) × 100%.

#### Statistical Methods

The median inhibitory concentration (IC_50_) was calculated using the probit analysis method in IBM SPSS statistics 22, and one-way ANOVA software GraphPad prism 6.02 was used for intergroup comparison at *p* < 0.05 level, which was considered to be statistically significant.

## Results

### Structural Determination of Compounds 2–4

Compound **2** is a light-yellow powder with solubility in chloroform, methanol, ethyl acetate, and other lower polar solvents. Its molecular formula of C_29_H_38_O_5_ was determined using the HRESIMS data of the ions as [M–H]^–^ at *m/z* 465.2720, in combination with the NMR data analysis. The ^1^H NMR (Table 1) that was acquired in CD_3_OD showed resonances, including six singlet methyl groups at δ_H (ppm)_ 0.78 (s, H_3_-27), 1.27 (s, H_3_-28), 1.30 (s, H_3_-29), 1.47 (s, H_3_-25), 1.56 (s, H_3_-26), and 2.21 (s, H_3_-23). A group of proton peaks in the downfield region of 6.44 (d, *J* = 1.2 Hz, H-1), 7.23 (dd, *J* = 7.2, 1.2 Hz, H-6), and 6.54 (d, *J* = 7.2 Hz, H-7) resembled the resonances attributed to A and B rings in celastrol. Compared with celastrol, one additional oxymethine signal at δ_H_ 4.00 (dd, *J* = 8.8, 6.8 Hz, H-16) indicated that compound **2** could be a hydroxylation product of compound **1.** This was also supported by the 16 Da difference in mass number between compound **2** and **1**. The comparison of ^13^C NMR (Table 2) between compounds **2** and **1** indicated that one methylene (CH_2_, at 36.1 δ_C_) in compound **1** was transformed to oxymethine (CH-O, at δ_C_ 74.0 ppm) in compound **2**, which is consistent with the ^1^H NMR hypothesis.

**TABLE 1:** ^1^H NMR spectroscopic data for compounds **1**–**3** in CD_3_OD and **4** in CDCl_3_^a^.

No.	1	2	3	4^b^	1^c^
1	6.46, d (1.2)	6.44, d (1.2)	6.68 s	6.51 s	6.50, d (1.2)
6	7.21, dd (7.2, 1.2)	7.23, dd (7.2,1.2)	a: 3.40, dd (20.4, 5.6)	7.06, d (7.6)	7.07 dd (7.2, 1.2)
			b: 3.02, brd (20.4)		
7	6.47, d (7.2)	6.54, d (7.2)	5.91, brd (5.6)	6.34, d (7.6)	6.34, d (7.2)
11	2.21, m	a: 1.55, m	2.04, m	a: 1.82, m	–
	1.90, m	b: 2.18, m		b: 1.66, m	
12	1.88, m	a: 1.80, m	a:1.98, m	a: 2.13, m	–
	1.29, m	a: 1.88, m	b:1.56, m	b: 1.65, m	
15	2.12, m	a: 2.41, brdd (14.4, 8.8)	a:2.45, brdd (14.4, 9.2)	a: 1.77, m	–
	1.73, m	b: 1.77, brdd (14.4, 6.8)	b:1.68, brdd (14.4, 8.0)	b: 1.29, m	
16	2.13, m	4.00, dd (8.8, 6.8)	4.03, dd (9.2, 8.0)	2.08, 0.93, m	–
	0.93, m				
18	1.66, m	1.80, m	1.80, m	1.58, m	–
19	a: 2.47, brd (15.6)	a: 2.07, dd (14.4, 6.8)	a: 1.75, m	a: 2.48, d (16.0)	–
	b:1.73, dd (15.6, 7.2)	b: 1.59, m	b: 1.68, m	b: 1.73, d (16.0)	
21	1.66, 2H, m	2.16–2.24, m	a: 2.20, m	a: 1.61, m	–
			b: 1.45, m	b: 1.31, m	
22	1.51, m	a: 1.50, m	1.55, m	a: 1.50, m	–
	1.93, m	b: 1.87, m		b: 1.87, m	
23	2.21, s	2.21, s	2.15, s	2.22, s	2.22, s
25	1.46, s	1.47, s	1.28, s	1.44, s	1.44, s
26	1.19, s	1.56, s	1.53, s	1.22, s	1.25, s
27	0.73, s	0.78, s	0.81, s	0.60, s	0.58, s
28	1.13, s	1.27, s	1.27, s	1.09, s	1.10, s
29	1.31, s	1.30, s	1.32, s	1.24, s	1.29, s

*^a^ ^1^H NMR data (δ) was measured at 400 MHz in CD_3_OD (**1–3**) and CDCl_3_ (**4**). Proton coupling constants (J) in Hz are given in parentheses. The assignments were based on DEPT, ^1^H-^1^H COSY, gHMQC, and HMBC experiments. ^b^ Data for the 7,9-octadecadienoic acyl group were provided as H-2’: 2.33, 2.14, m; H-3’: 2.33, 1.62, m; H-4’: 1.23, 1.84, m; H-5’: 2.05, m; H-6’: 2.78, m; H-7’: 5.30-5.43, m; H-8’: 5.30-5.43, m; H-9’: 5.30-5.43, m; H-10’: 5.30-5.43, m; H-11’: 2.05, m; H-12’: 2.23, 1.36, m; H-13’: 1.87, 1.36, m; H-14’: 1.54, m; H-15’: 1.61, 1.31, m; H-16’: 1.30, 0.89, m; H-17’: 1.31, m; H-18’: 0.89, t, J = 7.2 Hz. ^c^ Data of **1** in CDCl_3_ were also provided as ref.*

**TABLE 2:** ^13^C NMR spectroscopic data for compounds 1–3 in CD_3_OD and 4 in CDCl_3_^a^.

No.	1	2	3	4^b^	1^c^
1	119.9	118.8	109.1	120.5	120.7
2	180.2	178.8	144.5	178.5	178.5
3	148.0	146.3	141.8	147.0	147.2
4	120.6	119.2	122.1	119.9	120.7
5	128.8	127.3	125.9	127.7	127.8
6	136.6	134.7	29.0	135.2	135.7
7	120.3	118.9	121.1	118.5	118.5
8	172.4	172.0	153.5	172.3	173.0
9	44.4	43.2	44.4	43.3	43.3
10	166.6	164.4	141.8	165.2	165.3
11	34.8	32.2	35.2	33.9	34.0
12	30.8	29.8	32.3	29.8	29.5
13	41.4	39.3	38.1	40.2	40.1
14	46.5	44.3	39.4	45.5	45.5
15	31.8	39.8	43.6	31.7	30.9
16	36.1	74.0	76.3	34.7	34.7
17	31.0	35.9	37.2	30.8	29.7
18	45.8	43.8	45.5	44.5	44.7
19	32.1	30.8	32.1	31.2	31.3
20	40.8	39.8	41.3	39.5	39.5
21	29.9	28.3	29.7	29.2	28.9
22	37.7	35.7	37.4	36.6	36.6
23	10.5	8.8	11.8	10.6	10.7
25	39.1	38.1	33.9	38.5	38.6
26	33.3	26.8	30.2	32.7	32.6
27	19.8	19.8	21.6	18.9	18.9
28	32.2	24.6	26.2	31.7	31.7
29	22.4	26.4	27.1	21.7	21.7
30	182.6	182.6	183.8	183.3	182.5

*^a^ ^13^C NMR data (δ) was measured at 400 MHz in CD_3_OD (**1–3**) and CDCl_3_ (**4**). The assignments were based on DEPT, ^1^H-^1^H COSY, gHMQC, and HMBC experiments, as well as compared to those of celastrol in literature. ^b^ Data for the 7,9-octadecadienoic acyl group were provided as C-1’: 179.1; C-2’: 34.1; C-3’: 24.9; C-4’: 29.4; C-5’: 27.4; C-6’: 25.8; C-7’: 128.1; C-8’: 130.3; C-9’: 128.3; C-10’: 130.4; C-11’: 27.4; C-12’: 29.7; C-13’: 29.9; C-14’: 28.9; C-15’: 29.3; C-16’: 22.8; C-17’: 29.6; C-18’: 14.3. ^c^ Data of **1** in CDCl_3_ were also provided as ref.*

**FIGURE 2:**
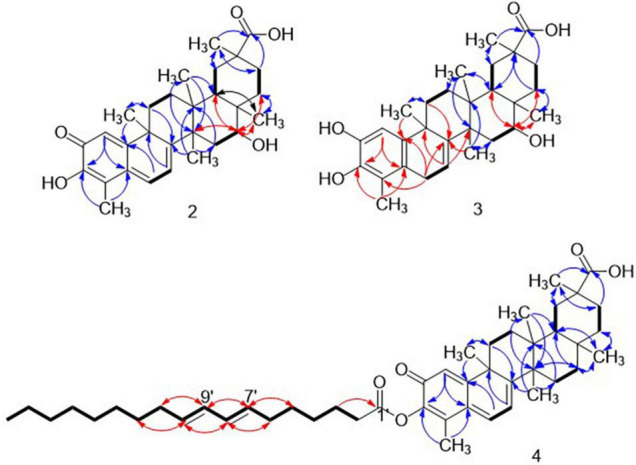
Key ^1^H–^1^H COSY (—) and HMBC (H→C) correlations of compounds **2**–**4**. HMBC correlations for determining differential structure fragments to compound **1** were assigned in red arrows, while the same correlations were assigned in blue arrows.

The proton and proton-bearing carbon signals in the NMR spectra of compound **2** were assigned using the gHMQC experiment. In the ^1^H-^1^H COSY spectrum of compound **2** (Figure 2), the cross-peaks of (CH)_sp2_-(CH)_sp2_; 2 × CH_2_-CH_2_; CH_2_-CH-O; and a CH-CH_2_, together with ^13^C NMR and DEPT data, indicated that compound **2** was a novel quinone methide pentacyclic triterpenoid, which closely resembled celastrol. The additional oxymethine was determined to be at C-16 on basis of the HMBC correlations from H-16 to C-14, C-18, C-22, and CH_3_-28, and from H-18, H_2_-22, and H_3_-28 to C-16 (Figure 2), as well as the NMR shifts around CH-16, such as Δδ_C–15_ + 8 ppm; Δδ_C–17_ + 4.9 ppm; and Δδ_C–18_ –2.0 ppm.

**FIGURE 3:**
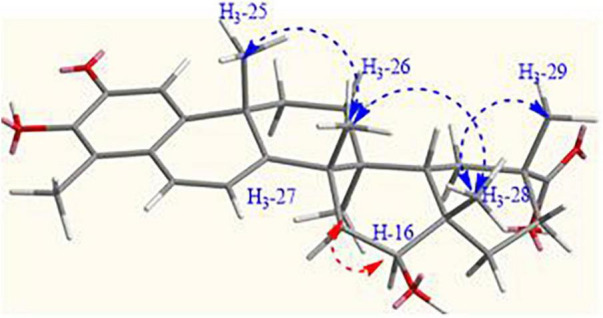
NOESY correlations (H↔H) of **2**.

The relative configuration of CH-16 was determined using the NOESY experiment. The correlations of H_3_-25/H_3_-26, H_3_-26/H_3_-28, and H_3_-28/H_3_-29 indicated that these methyl groups remained on one side of the plane, identical to compound **1**, while the correlation of H-16 with H_3_-27 indicated that they were oriented on the other side (Figure 3). Therefore, OH-16 was in the β orientation. As compound **2** was generated from the mono oxidation of compound **1**, the absolute configuration of **1** and its derivatives were determined multiple times using X-ray crystal analysis (Zha et al., 2018). Based on the results of the above relative configuration analysis and the conservation of the configuration of natural products from the same species, the absolute configuration of C-16 was determined as *S-*configuration. Therefore, compound **2** was finally determined as *S*-16-hydroxyl celastrol.

The retention time for compound **3** peaks at 24.3 min was shorter than for compound **2** at 26.8 min. The negative HRESIMS of the quasi-molecular ion at *m/z* 467.3026 [M–H]^–^ indicated the molecular formula of C_29_H_40_O_5_, 2 Da larger than that of **2**. The ^1^H NMR data (Table 1) gave the same oxymethine signal at δ_H_ 4.03, dd, (9.2, 8.0), and similar resonances belonging to six singlet methyl groups at δ_H_ 0.81 (H_3_-27); 1.27 (H_3_-28); 1.28 (H_3_-25); 1.32 (H_3_-29);1.53 (H_3_-26); and 2.15 (H_3_-23) in comparison with compound **2**. All these similar ^1^H NMR signals suggest that compound **3**, just like compound **2**, is also a mono oxygenated product of celastrol. Detailed analysis of the ^1^H NMR of compound **3** indicated that the AA’ coupled system of H-6/H-7 (7.8 Hz) in compound **2** was transformed to A_2_X system at δ_H (ppm)_ 3.40 (dd, *J* = 20.4, 5.6 Hz, H-6a), 3.02 (brd, *J* = 20.4 Hz, H-6b), and 5.91 (brd, *J* = 5.6 Hz). The 0.24 ppm downfield shifted signal of H-1, and 0.63 ppm upfield shifted proton of H-7 suggested that the changes occurred around A and B rings. The upfield carbon resonances of C-1, C-2, C-3, C-5, C-6, C-8, and C-10 were Δδ_C_ –9.7, –34.3, –4.5, –1.4, –105.7, –18.5, and –22.6 ppm, respectively, while the downshield carbon resonances of C-4, C-7, and C-9 were Δδ_C_ + 2.9, + 2.2, and + 1.2 ppm, respectively (Table 2). Comparison of the ^13^C NMR data of compound **3** with **2** indicated that the characteristic quinone methide structure in **1** had disappeared and was transformed into a 1,4-dihydronaphthyl group that has been generated by chemical modification (Figueiredo et al., 2017). Therefore, the structure of **3** could be described as *S*-16-hydroxyl dihydrocelastrol.

The method of determining the stereo-configuration of the oxidation site of C-16 in **3** was the same as that of compound **2**. The relative configuration of H_3_-25/H_3_-26, H_3_-26/H_3_-28, and H_3_-28/H_3_-29 was opposite to H-16 and H_3_-27 that could be deduced to the NOESY correlations. Moreover, the absolute configuration of C-16 was maintained as *S*-configuration. Thus, compound **3** was finally determined as *S*-16-hydroxyl dihydrocelastrol.

The (-)-HRESIMS at *m/z* 711.6245 [M–H]^–^ of **4** indicated the molecular formula of C_47_H_68_O_5_, 262 Da larger than that of compound **1**, indicating that a chemical fragment had been coupled to celastrol by condensation. The ^1^H NMR of compound **4** gave almost all of the proton resonances corresponding to compound **1,** including six singlet methyl groups, three sp^2^ hybridized methines, and some saturated methylene and methine groups. Besides, the additional signals at δ_H (ppm)_ 0.89 (t, *J* = 7.2 Hz, H-18’) and four coupled methines proton resonances at δ_H (ppm)_ 5.30–5.43, in combination with the chemical shift region of 0.9∼2.5 ppm, attributed to saturated groups, indicating that the additional chemical fragment could be an unsaturated fatty acyl. This suggestion was supported by the ^13^C and 2D NMR analysis. In ^13^C NMR spectrum, the additional 18 carbon resonances, including one methyl, 12 methylene, four sp^2^ hybridized methines, and one carbonyl (179.1 ppm) indicated that compound **4** could be a octadecadienoic acid ester of celastrol. Further 2D NMR analysis including ^1^H-^1^H COSY and HMBC analysis of compound **4** (Figure 2) showed that the diene site of the fatty acyl is located at C7’ to C-10’ as a butadienyl, through correlation of H_2_-6’/H-7’/H-8’/H-9’/H-10’/H_2_-11’. (^1^H-^1^H COSY) and H-7’ to C-5’ and C-9’, H-8’ to C-6’ and C-10’, H-9’ to C-7’ and C-11’ (HMBC). The slight chemical shift changes of C-1 (Δδ_C_), C-2 (Δδ_C_), C-3 (Δδ_C_), and C-4 (Δδ_C_) indicated that the 7,9-octadecadienoic acid was esterified at the 3-OH. Therefore, compound **4** was determined as 7,9-octadecadienoic acid ester of celastrol.

### The Species Identification of LGT-5

**FIGURE 4:**
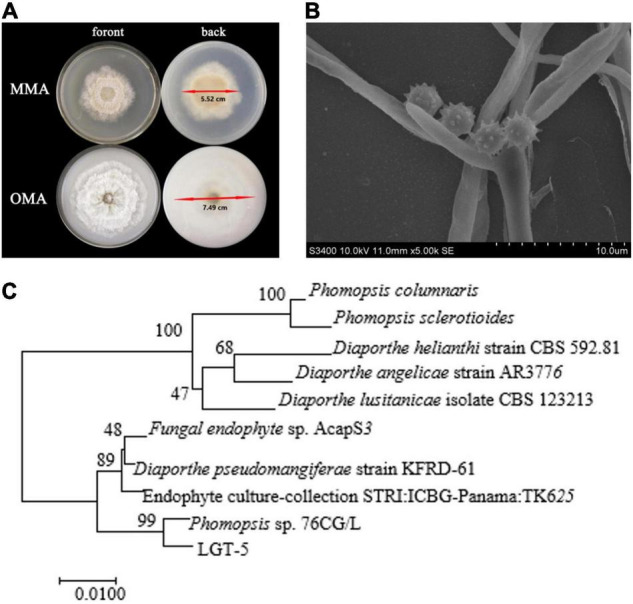
The images of strain growth, mycelium, and phylogenetic tree of *Phomopsis* sp. LGT-5. **(A)** Strain cultured with MMA and OMA medium; **(B)** Hyphae morphology from SEM scanning; **(C)** Evolutionary tree of *Phomopsis* sp. L.

The LGT-5 strain was isolated from fresh *T. wilfordii* Hook F. and was cultured in MMA and OMA (Motta and Santana, 2012). The diameter of bacterial colonies on MMA and OMA was 5.52 and 7.49 cm, respectively, after 5 days of growth (Figure 4A). Scanning electron microscopy could clearly distinguish the branch of the strain with a diameter of 2.0 μm (Figure 4B). The universal primers ITS1 (TCCGTAGGTGAACCTGCGG) and ITS4 (TCCTCCGCTTATTGATATGC) were used for PCR amplification. Sequence homology analysis with ITS-18S (Figure 4C) indicated that LGT-5 resembled *Phomopsis* sp. 76CG/L. Consequently, LGT-5 was named as *Phomopsis* sp. LGT-5, and was deposited into the China General Microbiological Culture Collection Center (CGMCC No. 16088).

### The Results of Cytotoxicity Assay

Compounds **2**–**4** were screened for antitumor activities against U251, A549, KG-1, B16 cell lines and for cytotoxicity against BV-2, H9c2, and PC12 cell lines (Table 3 and Supplementary Figures 1–6). The activities for compound **3** and **4** against tumor cells U251, A549, KG-1, and B16 were similar to celastrol, while the activity of compound **2** declined almost 10 times. When compared with celastrol, the cytotoxicity of compounds **2**–**4** declined 11 to 31-folds against BV-2, but compound **2** and **3** had identical cytotoxicity against H9c2 and PC12 cell lines with celastrol. The abovementioned results indicated that the addition of 16*S* -OH group in compound **2** and **3** might play a key role in reducing the cytotoxicity, while the destruction of the conjugate system of A and B ring in compound **2** and 7, 9-octadecadienoic acid esterification substituted with 3-OH in compound **4** may also be important factors to improve antitumor activity and reduce their side effects.

**TABLE 3:** IC_50_ values (μM) of **2**–**4** against U251, A549, KG-1, B16, BV-2, H9c2, and PC12 cell lines (IC_50_, μM, *n* = 3).

	Celastrol	2	3	4
U251	3.36	43.87	8.12	1.69
A549	4.38	27.03	6.51	4.47
KG-1	4.35	23.21	2.61	3.50
B16	4.35	12.19	13.10	10.13
BV-2	3.04	92.82	34.25	74.75
H9c2	5.39	15.36	7.60	–
PC12	4.85	8.05	5.75	–

*–, undetected.*

## Discussion

According to incomplete statistics, more than 50% of small molecule drugs in clinical application are directly or indirectly derived from natural products, among which the molecules from microorganisms are used in antibacterial, antiviral, antitumor, and other aspects due to their structural diversity and unique biological activities (Newman and Cragg, 2020). For example, rapamycin, generated by *S. hygroscopicus* is used as an immunosuppressant in renal transplantation (Vignot et al., 2005; Yoo et al., 2017). Romidepsin is a histone deacetylase inhibitor with antitumor activity, which is used in clinical treatment of T-cell lymphoma. It was first isolated from Gram-negative bacteria *Chromobacterium violaceum* (VanderMolen et al., 2011). Geldanamycin is also derived from *S. hygroscopicus* (Díaz-Cruz et al., 2022), a kind of benzoxazole antibiotic; it was found to have antiparasitic and antitumor activities in early studies. But due to poor stability and hepatotoxicity, preclinical studies have been discontinued. A derivative of geldanamycin (IPI-504) is still in phase II clinical trial (Di et al., 2014). Among the 22 kinds of antibacterial drugs on the market since 2000, 12 of them come from microbial secondary metabolites (Newman and Cragg, 2020). All these examples show that microorganisms play a very important role in the development of new drugs. In the past, the research of natural drugs mainly focused on the discovery of secondary metabolites and the evaluation of biological activity, as well as the chemical modification based on the original compounds, but ignored the biological modification of natural products. Celastrol is one of the most classical natural products with cytotoxic activity, but because of its structural characteristics, it is unable to obtain a variety of chemical modification products for further pharmaceutical research. This study introduces a new method to study celastrol. It overcomes the serious toxicity of celastrol by using endophyte as a biotransformation strain, and obtain new structural derivatives. The method in this study opens another door for the study of celastrol, and will also provide reference for the study of other similar drugs.

## Conclusion

This study described a method of microbial transformation to improve the modification site of celastrol and reduce its toxicity. LGT-5 resembled *Phomopsis* sp. 76CG/L. in sequence homology analysis with ITS-18S that from *T. wilfordii* Hook F., the native plant of celastrol, and showed strong toxicity resistance against celastrol. After co-culture with LGT-5, celastrol was bio-transformed into novel derivatives. Based on rapid isolation and structural identification, we reported three new compounds (**2**–**4**) with reduced toxicity and structural diversity. The 16*S*-OH derivatives of **2** and **3** increased the modification site for further chemical derivatization.

## Data Availability Statement

The original contributions presented in the study are included in the article/Supplementary Material, further inquiries can be directed to the corresponding authors.

## Author Contributions

X-LW and Z-BJ are responsible for the overall arrangements for the study. P-YM and W-LG made substantial contributions to the experimental operation and data acquisition. P-YM drafted the manuscript. JC revised the manuscript critically for important intellectual content. H-YJ, CL, B-WY, and SW analyzed and interpreted the data. All authors read and approved the final manuscript.

## Conflict of Interest

The authors declare that the research was conducted in the absence of any commercial or financial relationships that could be construed as a potential conflict of interest.

## Publisher’s Note

All claims expressed in this article are solely those of the authors and do not necessarily represent those of their affiliated organizations, or those of the publisher, the editors and the reviewers. Any product that may be evaluated in this article, or claim that may be made by its manufacturer, is not guaranteed or endorsed by the publisher.

## Supplementary Material

The HRESIMS and NMR spectra for compounds **1**–**4**, and the ITS-18S sequencing of LGT-5 were provided in the Supplementary Appendix A.

## Abbreviations

1D and 2D NMR: one-Dimensional and two-Dimensional Nuclear Magnetic Resonance
TLC: Thin Layer Chromatography
IC_50_: 50% Inhibiting Concentration
NOESY: Nuclear Overhauser Effect Spectroscopy
HPLC: High Performance Liquid Chromatography
RP-HPLC: Revise Phase, High Performance Liquid Chromatography
HRESIMS: High Resolution Electrospray Ionization Mass Spectrometry
DEPT: Distortionless Enhancement by Polarization Transfer
^1^H-^1^H COSY: Homonuclear Chemical Shift Correlation Spectroscopy
HMQC: Heteronuclear Multiple Quantum Correlation
HMBC: Heteronuclear Multiple Bond Correlation.

## References

[B1] Chen S. R. Dai Y. Zhao J. Lin L. Wang Y. Wang Y. (2018). A mechanistic overview of triptolide and celastrol, natural products from *Tripterygium wilfordii* Hook F. *Front. Pharmacol.* 9:104. 10.3389/fphar.2018.00104 29491837 PMC5817256

[B2] Cheng Y. Y. Yang X. Gao X. Song S. X. Yang M. F. Xie F. M. (2021). LGR6 promotes glioblastoma malignancy and chemoresistance by activating the Akt signaling pathway. *Exp. Ther. Med.* 22 1364–1373. 10.3892/etm.2021.10798 34659510 PMC8515564

[B3] de Souza L. Ferreira F. U. Thome C. H. Brand H. Orellana M. D. Faça V. M. (2021). Human and mouse melanoma cells recapitulate an EMT-like program in response to mesenchymal stromal cells secretome. *Cancer Lett.* 501 114–123. 10.1016/j.canlet.2020.12.030 33383153

[B4] Di K. Keir S. T. Alexandru-Abrams D. Gong X. Nguyen H. Friedman H. S. (2014). Profiling Hsp90 differential expression and the molecular effects of the Hsp90 inhibitor IPI-504 in high-grade glioma models. *J. Neurooncol.* 120 473–481. 10.1007/s11060-014-1579-y 25115740

[B5] Díaz-Cruz G. A. Liu J. Tahlan K. Bignell D. R. (2022). Nigericin and geldanamycin are Phytotoxic specialized metabolites produced by the plant pathogen *Streptomyces sp.* 11-1-2. *Microbiol. Spectr.* 2022 2314–2321. 10.1128/spectrum.02314-21 35225656 PMC9045263

[B6] Figueiredo S. A. C. Salvador J. A. R. Cortes R. Cascante M. (2017). Novel celastrol derivatives with improved selectivity and enhanced antitumour activity: design, synthesis and biological evaluation. *Eur. J. Med. Chem.* 138 422–437. 10.1016/j.ejmech.2017.06.029 28688281

[B7] He Q. W. J. Feng H. Hu X. L. Long H. Huang X. F. Jiang Z. Z. (2020). Synthesis and biological evaluation of celastrol derivatives as potential immunosuppressive agents. *J. Nat. Prod.* 83:2578. 10.1021/acs.jnatprod.0c00067 32822186

[B8] Hou W. Liu B. Xu H. T. (2020). Celastrol: progresses in structure-modifications, structure-activity relationships, pharmacology and toxicology. *Eur. J. Med. Chem.* 189:112081. 10.1016/j.ejmech.2020.112081 31991334

[B9] Hu L. G. Sun Y. K. Zhang H. S. Zhang J. G. Hu J. (2016). Catalpol inhibits apoptosis in hydrogen peroxide-induced cardiac myocytes through a mitochondrial-dependent caspase pathway. *Biosci. Rep.* 36:e00348. 10.1042/BSR20160132 27166426 PMC5293554

[B10] Jiang F. Wang H. J. Bao Q. C. Wang L. Jin Y. H. Zhang Q. (2016). Optimization and biological evaluation of celastrol derivatives as Hsp90-Cdc37 interaction disruptors with improved druglike properties. *Bioorg. Med. Chem.* 24 5431–5439. 10.1016/j.bmc.2016.08.070 27647369

[B11] Li N. Xu M. Y. Wang B. Shi Z. X. Zhao Z. H. Tang Y. Q. (2019). Discovery of novel celastrol derivatives as Hsp90-Cdc37 interaction disruptors with antitumor activity. *J. Med. Chem.* 62 10798–10815. 10.1021/acs.jmedchem.9b01290 31725288

[B12] Li P. Su R. X. Yin R. Y. Lai D. W. Wang M. A. Liu Y. (2020). Detoxification of mycotoxins through biotransformation. *Toxins* 12:121. 10.3390/toxins12020121 32075201 PMC7076809

[B13] Li X. Zheng Y. (2020). Biotransformation of lignin: mechanisms, applications and future work. *Biotechnol. Progr.* 36:e2922. 10.1016/B978-0-444-64279-0.00017-7 31587530

[B14] Liu J. Lee J. Salazar Hernandez M. A. Mazitschek R. Ozcan U. (2015). Treatment of obesity with celastrol. *Cell* 161 999–1011. 10.1016/j.cell.2015.05.011 26000480 PMC4768733

[B15] Liu Z. H. Dong M. Y. Qiu X. Y. Yin J. (2021). Diarylpentanones from the root of *Wikstroemia indica* and their cytotoxic activity against human lung A549 cells. *Nat. Prod. Res.* 35 3346–3349. 10.1080/14786419.2019.1698577 34590506

[B16] Mi E. K. Pu R. P. Ju Y. N. Inae J. Jun H. C. Jun S. L. (2019). Anti-neuroinflammatory effects of galangin in LPS-stimulated BV-2 microglia through regulation of IL-1β production and the NF-κB signaling pathways. *Mol. Cell Biochem.* 451 145–153. 10.1007/s11010-018-3401-1 29995265

[B17] Motta F. L. Santana M. H. A. (2012). Biomass production from *Trichoderma viride* in nonconventional oat medium. *Biotechnol. Progr.* 28 1245–1250. 10.1002/btpr.1578 22736524

[B18] Naimi A. Entezari A. Hagh M. F. Hassanzadeh A. Saraei R. Solali S. (2019). Quercetin sensitizes human myeloid leukemia KG-1 cells against TRAIL-induced apoptosis. *J. Cell Physiol.* 234 13233–13241. 10.1002/jcp.27995 30589076 PMC13483195

[B19] Newman D. J. Cragg G. M. (2020). Natural products as sources of new drugs over the nearly four decades from 01/1981 to 09/2019. *J. Nat. Prod.* 83 770–803. 10.1021/acs.jnatprod.9b01285 32162523

[B20] Qi X. Qin J. Ma N. Chou X. Wu Z. (2014). Solid self-microemulsifying dispersible tablets of celastrol: formulation development, charaterization and bioavailability evaluation. *Int. J. Pharm.* 472 40–47. 10.1016/j.ijpharm.2014.06.019 24929011

[B21] Shang F. F. Wang J. Y. Xu Q. Deng H. Guo H. Y. Jin X. J. (2021). Design, synthesis of novel celastrol derivatives and study on their antitumor growth through HIF-1α pathway. *Eur. J. Med. Chem.* 220:113474. 10.1016/j.ejmech.2021.113474 33930802

[B22] Shi J. F. Li J. X. Xu Z. Y. Chen L. Luo R. F. Zhang C. (2020). Celastrol: a review of useful strategies overcoming its limitation in anticancer application. *Front. Pharmacol.* 2020:741. 10.3389/fphar.2020.558741 33364939 PMC7751759

[B32] Sun H. Xu L. Yu P. Jiang J. Zhang G. Wang Y. (2010). Synthesis and preliminary evaluation of neuroprotection of celastrol analogues in PC12 cells. *Bioorg. Med. Chem. Lett.* 20, 3844–3847. 10.1016/j.bmcl.2010.05.066 20627556

[B23] Tang W. J. Wang J. Tong X. Shi J. B. Liu X. H. Li J. (2015). Design and synthesis of celastrol derivatives as anticancer agents. *Eur. J. Med. Chem.* 95 166–173. 10.1016/j.ejmech.2015.03.039 25812966

[B24] VanderMolen K. M. McCulloch W. Pearce C. J. Oberlies N. H. (2011). Romidepsin (Istodax, NSC 630176, FR901228, FK228, depsipeptide): a natural product recently approved for cutaneous T-cell lymphoma. *J. Antibiot.* 64 525–531. 10.1038/ja.2011.35 21587264 PMC3163831

[B25] Vignot S. Faivre S. Aguirre D. Raymond E. (2005). mTOR-targeted therapy of cancer with rapamycin derivatives. *Ann. Oncol.* 16 525–537. 10.1093/annonc/mdi113 15728109

[B26] Wang W. F. Wan Q. Li Y. X. Ge J. Feng F. Y. Yu X. Y. (2020). Application of an endophyte *Enterobacter sp.* TMX13 to reduce thiamethoxam residues and stress in Chinese cabbage (*Brassica chinensis* L). *J. Agr. Food Chem.* 68 9180–9187. 10.1021/acs.jafc.0c03523 32806115

[B27] Wu X. L. Liu C. Zhang C. F. Zhou L. Liu H. T. Wang S. (2020). *A New Compound and its Application in the Preparation of Antibacterial Agents. China Patent ZL201910145752.X.* Beijing: China National Intellectual Property Administration.

[B28] Yoo Y. J. Kim H. Park S. R. Yoon Y. J. (2017). An overview of rapamycin: from discovery to future perspectives. *J. Ind. Microbiol. Biot.* 44 537–553. 10.1007/s10295-016-1834-7 27613310

[B29] You D. Jeong Y. Yoon S. Y. Kim S. Kim S. W. Nam S. J. (2021). Celastrol attenuates the inflammatory response by inhibiting IL-1β expression in triple-negative breast cancer cells. *Oncol. Rep.* 45:89. 10.3892/or.2021.8040 33846813 PMC8042664

[B30] Zha J. Zhang Q. Li M. Q. Wang J. R. Mei X. F. (2018). Improving dissolution properties by polymers and surfactants: A case study of celastrol. *J. Pharm. Sci.* 107 2860–2868. 10.1016/j.xphs.2018.07.008 30017890

[B31] Zhang J. Li C. Y. Xu M. J. Wu T. Chu J. H. Liu S. J. (2012). Oral bioavailability and gender-related pharmacokinetics of celastrol following administration of pure celastrol and its related tablets in rats. *J. Ethnopharmacol.* 144 195–200. 10.1016/j.jep.2012.09.005 22982018

