# Supplementary material for: Native Endophytes of Tripterygium wilfordii-Mediated Biotransformation Reduces Toxicity of Celastrol
Source: Front Microbiol. 2022 May 25;13:810565. doi: 10.3389/fmicb.2022.810565 (PMC9177160; doi:10.3389/fmicb.2022.810565)
Supplement: Supplementary file 1 [file Data_Sheet_1.PDF]

Appendix A. Supplementary data

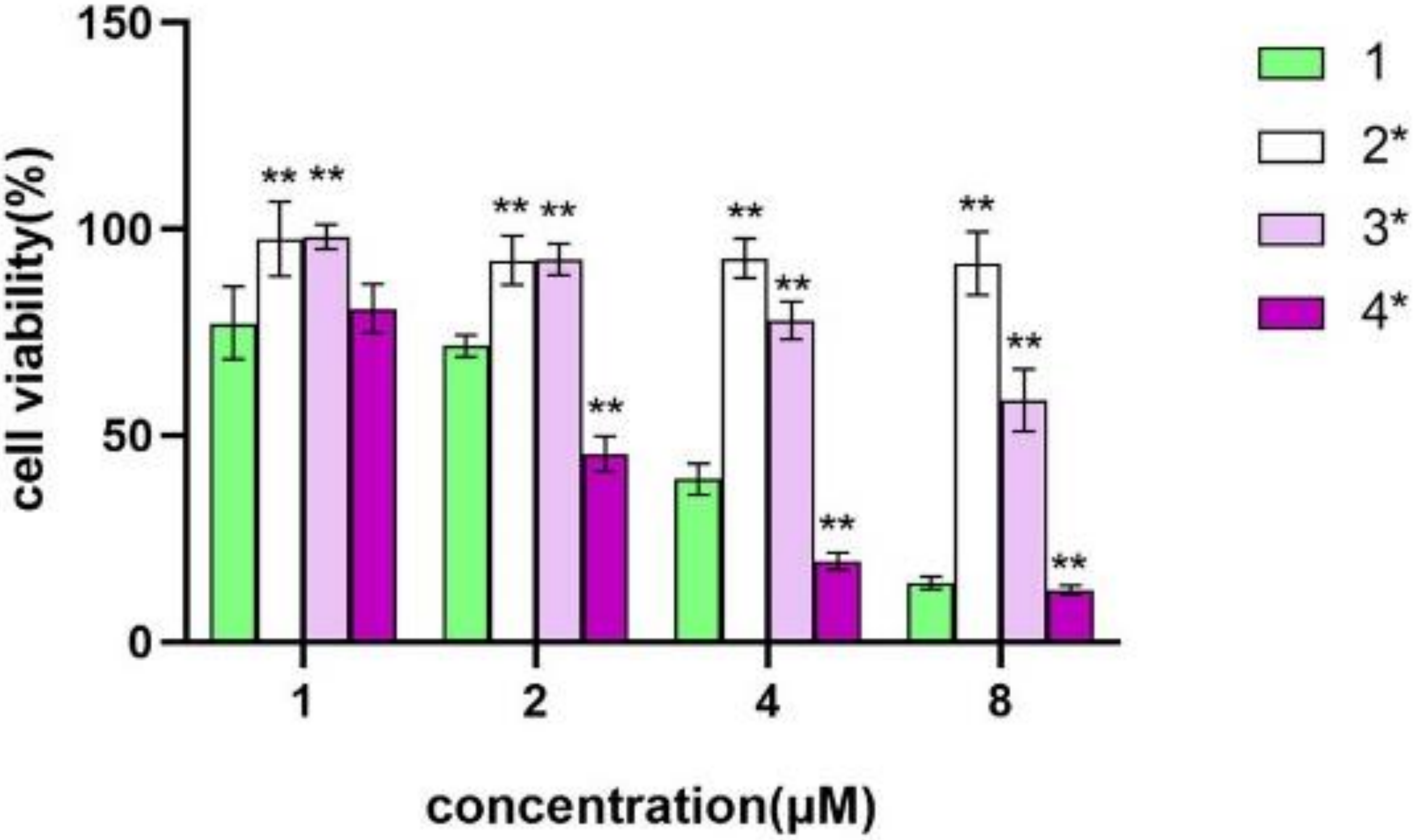

Figure S1 Effects of compounds **2-4** on cytotoxicity of glioma cells U251, comparing to celastrol (**1**). * $P < 0.05$, ** $P < 0.01$ (comparing to celastrol).

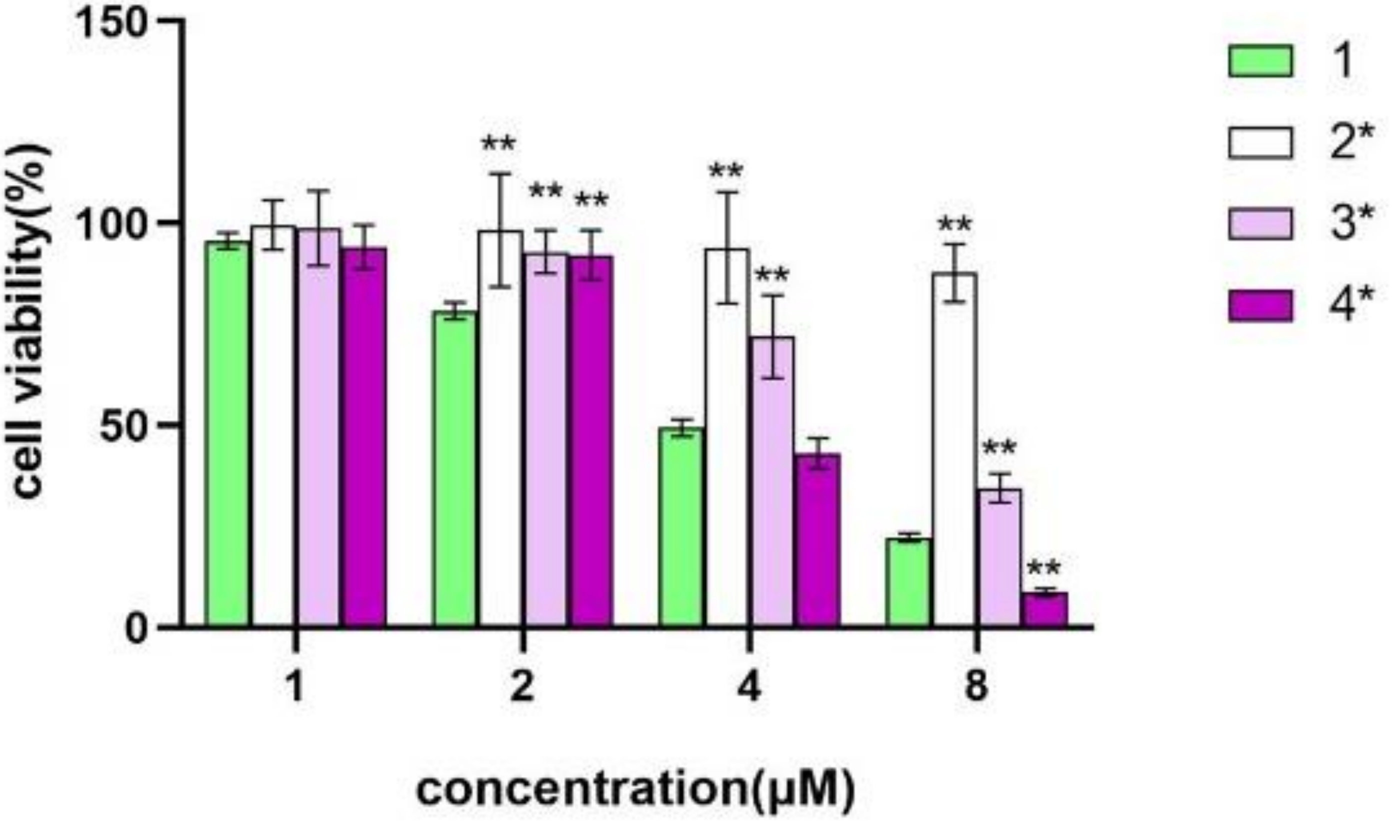

Figure S2 Effects of compounds **2**-**4** on cytotoxicity of lung cancer cell line A549, comparing to celastrol (**1**). $^{*}P < 0.05$, $^{**}P < 0.01$ (comparing to celastrol).

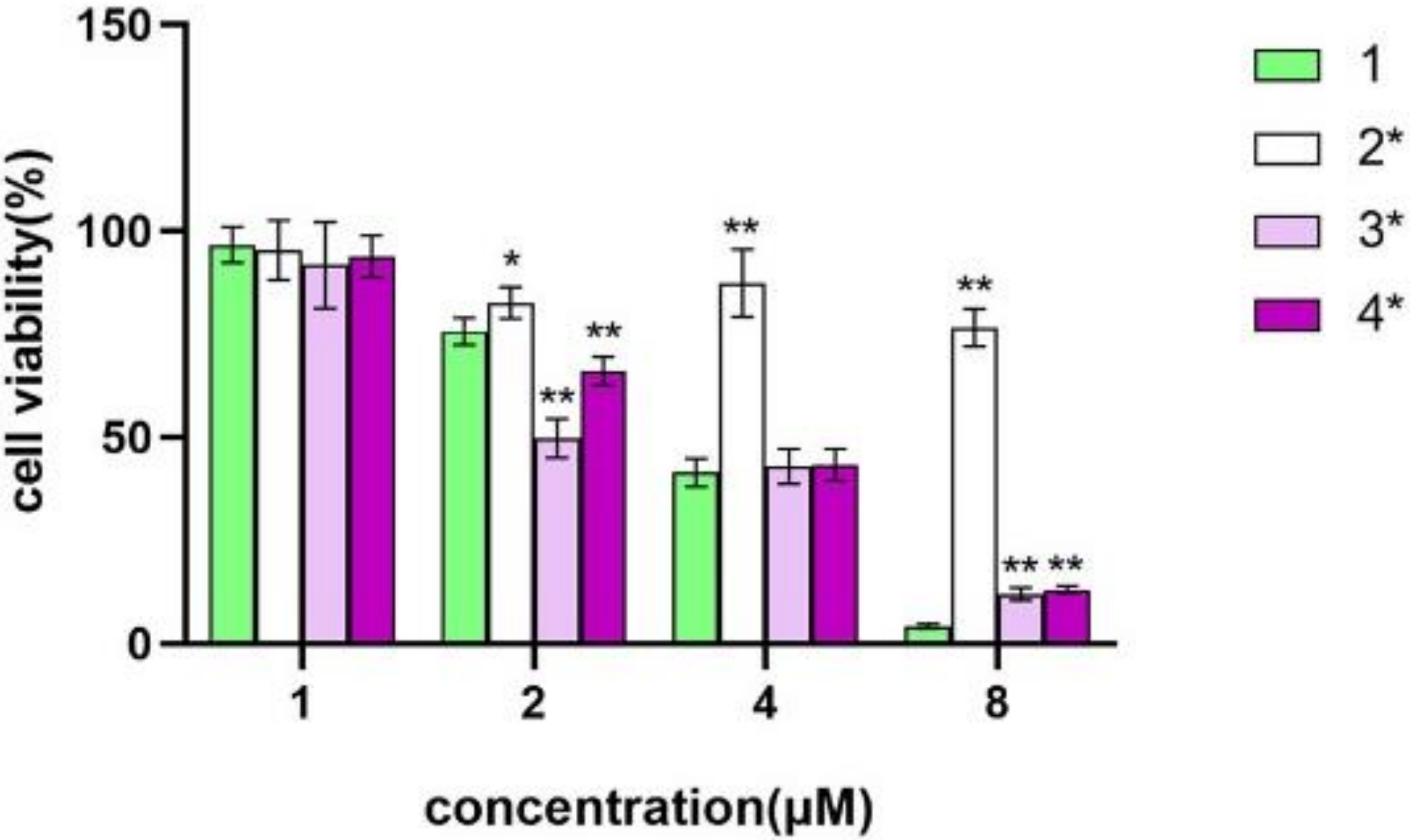

Figure S3 Effects of compounds **2-4** on cytotoxicity of acute myeloid leukemia cells KG-1, comparing to celastrol (**1**). * P < 0.05, ** P < 0.01 (comparing to celastrol).

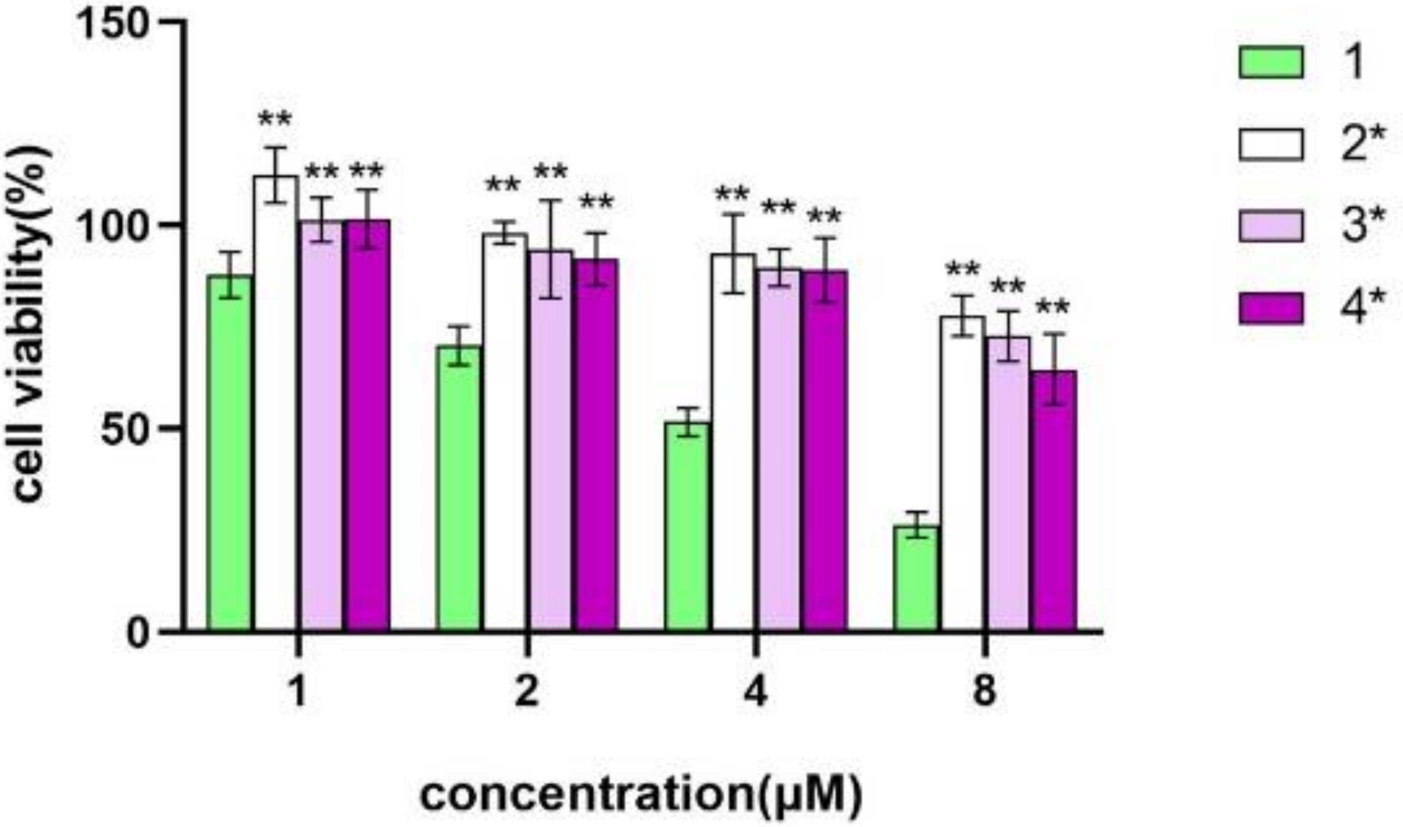

Figure S4 Effects of compounds **2-4** on cytotoxicity of mouse melanoma cells B16, comparing to celastrol (**1**). * $P < 0.05$, ** $P < 0.01$ (comparing to celastrol).

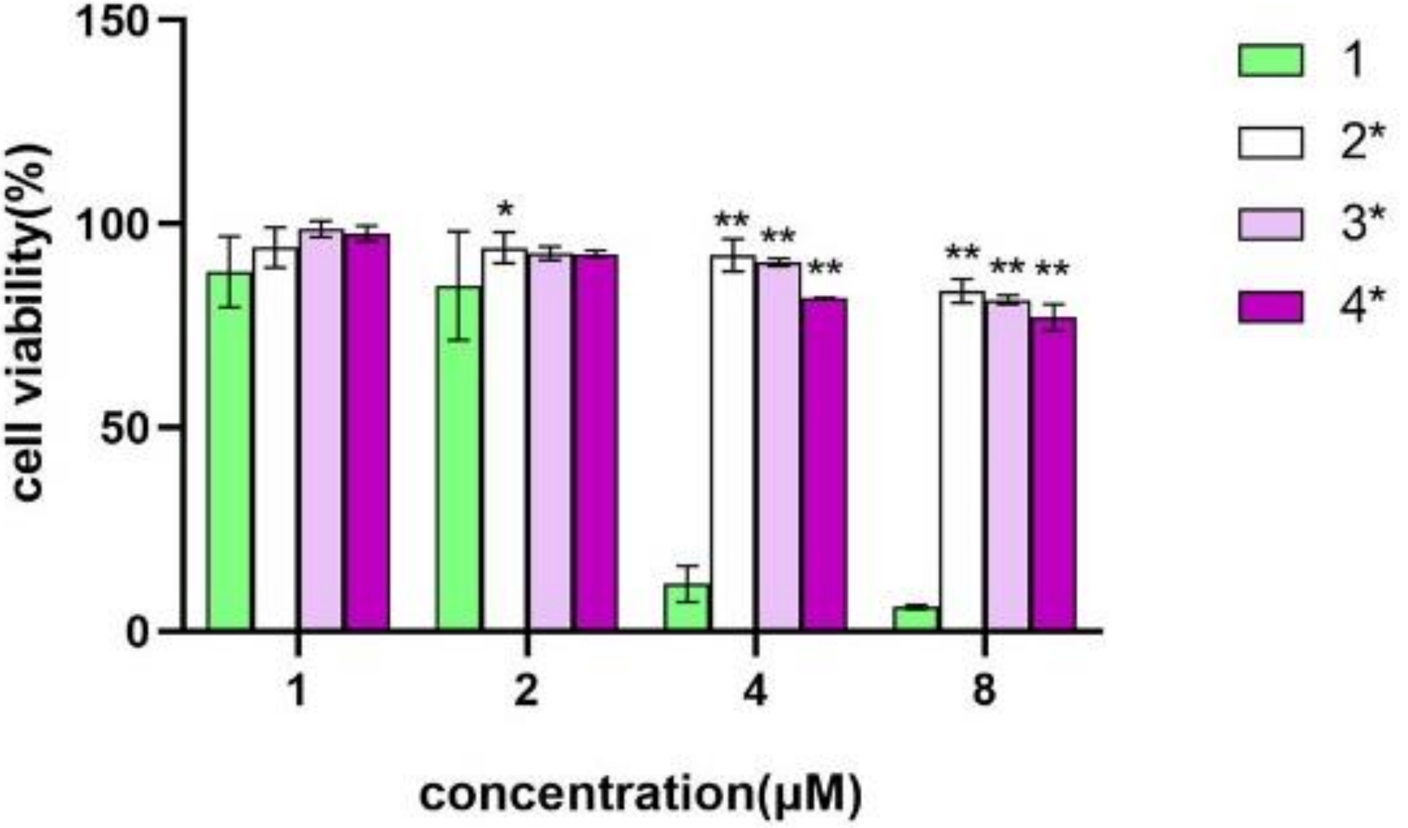

Figure S5 Effects of compounds **2-4** on cytotoxicity of BV-2, comparing to celastrol (**1**). * $P < 0.05$, ** $P < 0.01$ (comparing to celastrol).

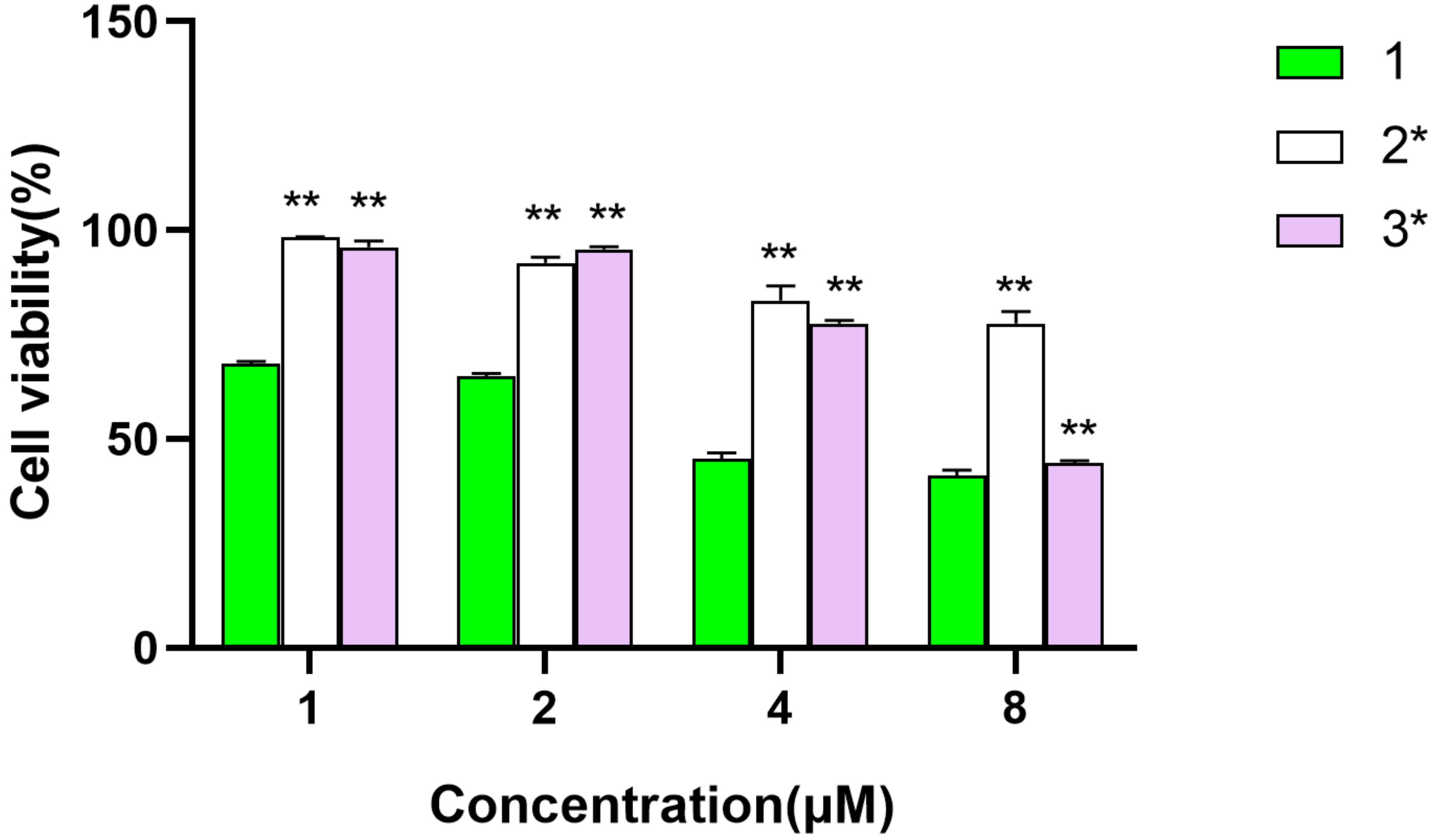

Figure S6 Effects of compounds **2** and **3** on cytotoxicity of H9c2, comparing to celastrol (**CSL**). ** $P < 0.01$ (comparing to celastrol)

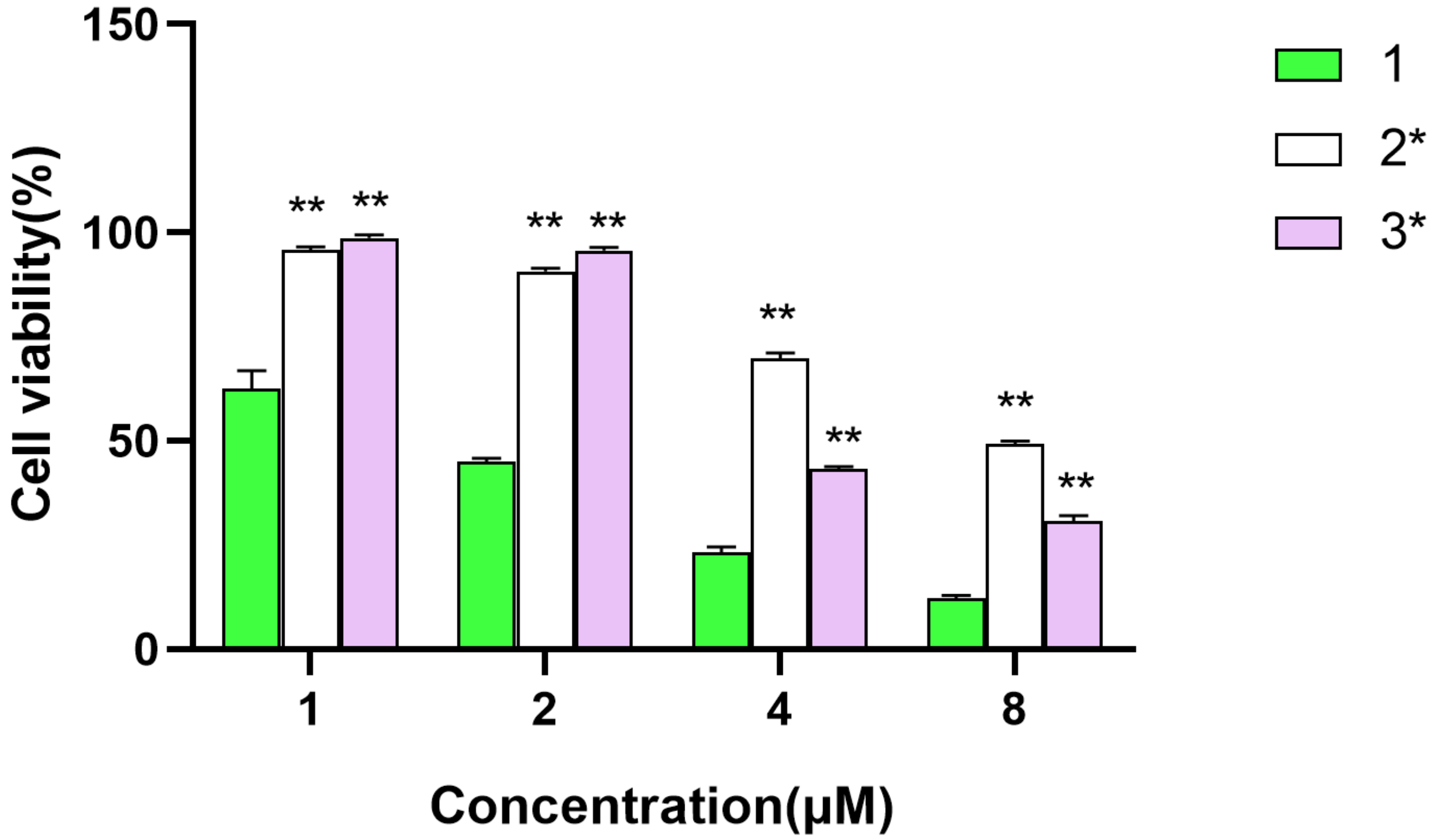

Figure S7 Effects of compounds **2** and **3** on cytotoxicity of PC12, comparing to celastrol (**CSL**). ** $P < 0.01$ (comparing to celastrol)

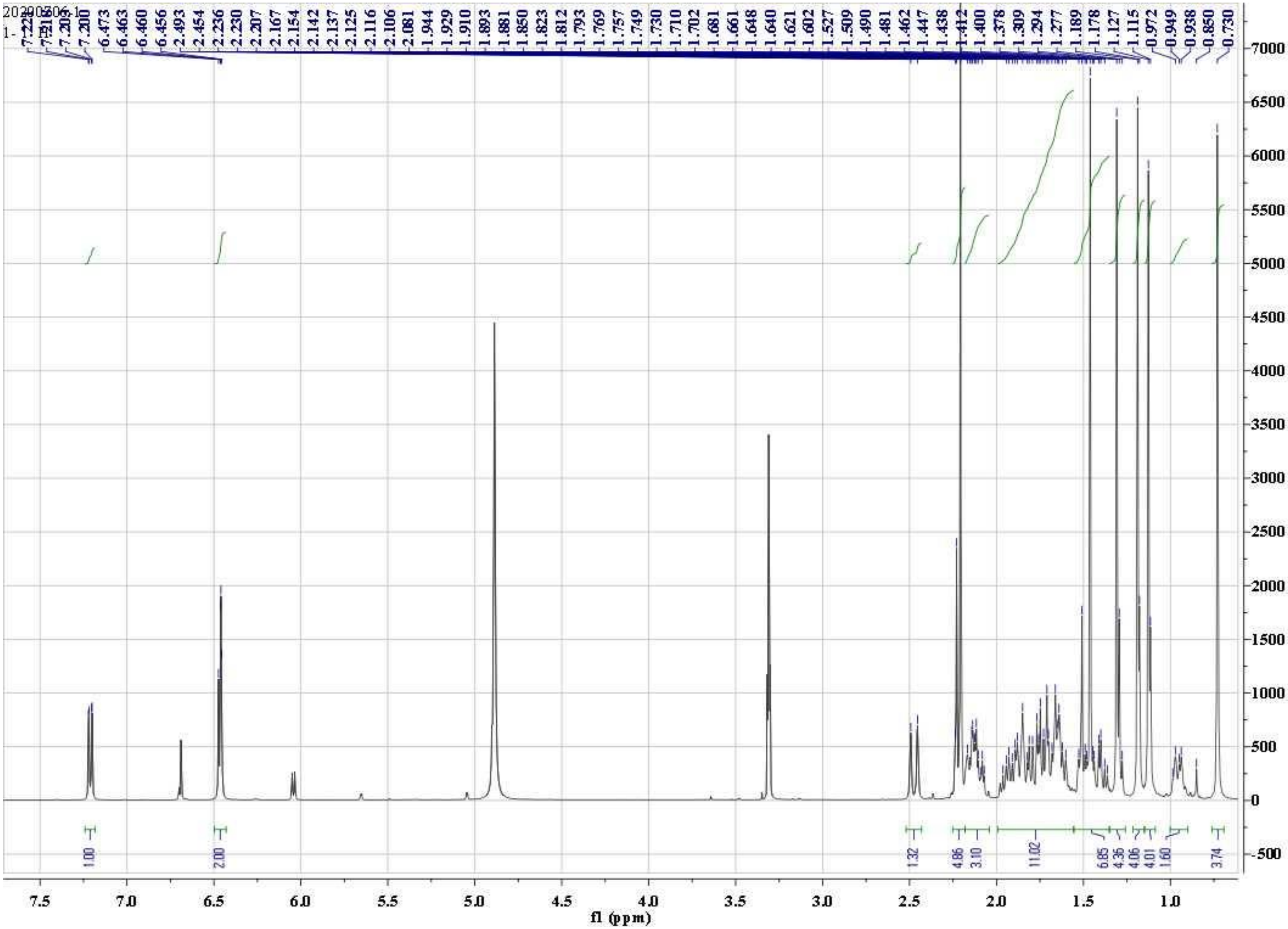

Figure S8 The ^{1}H NMR spectrum of celastrol (**1**) in CD_3OD at 400 MHz.

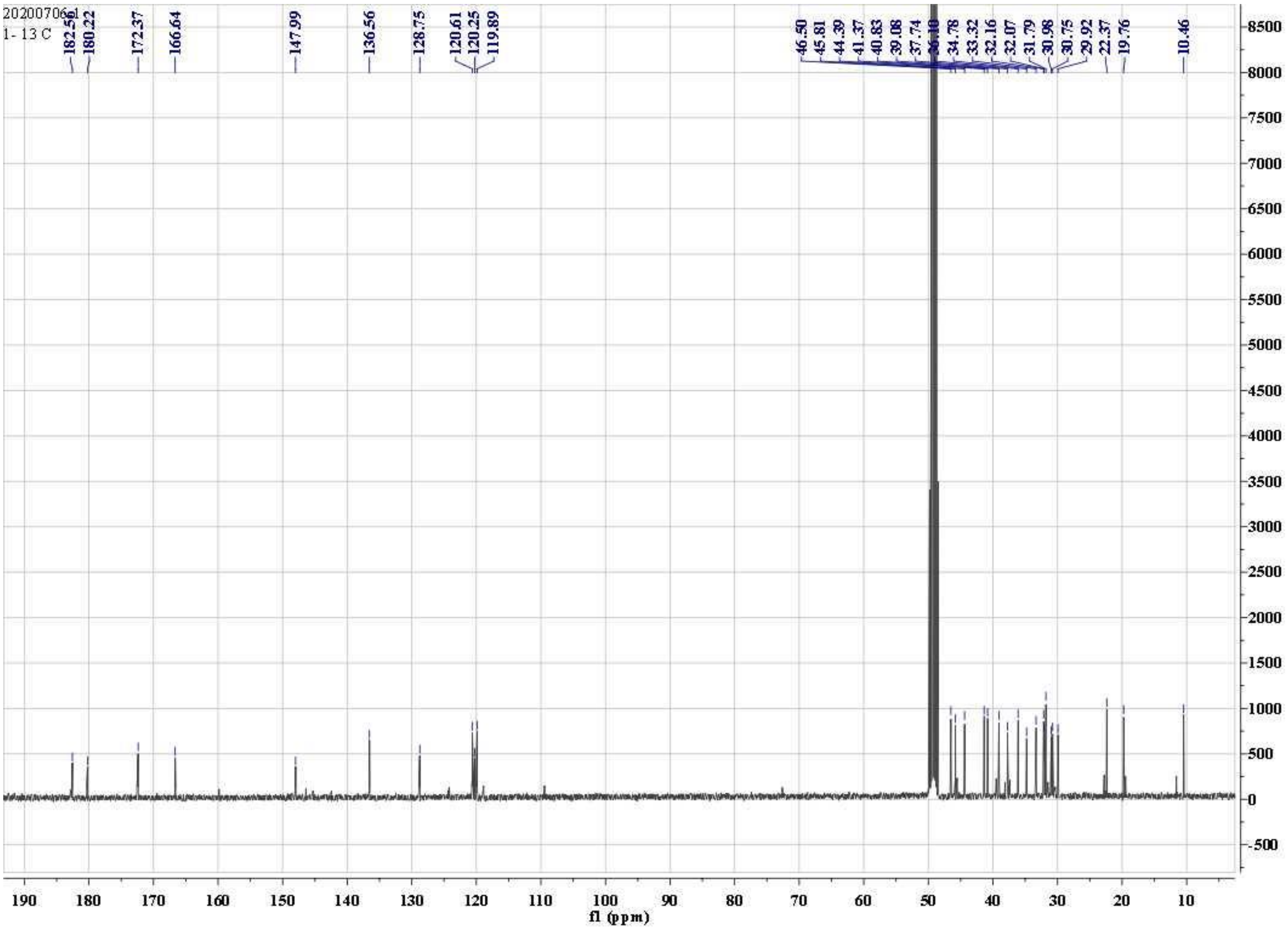

Figure S9 The ^{13}C NMR spectrum of celastrol (**1**) in CD_3OD at 100 MHz.

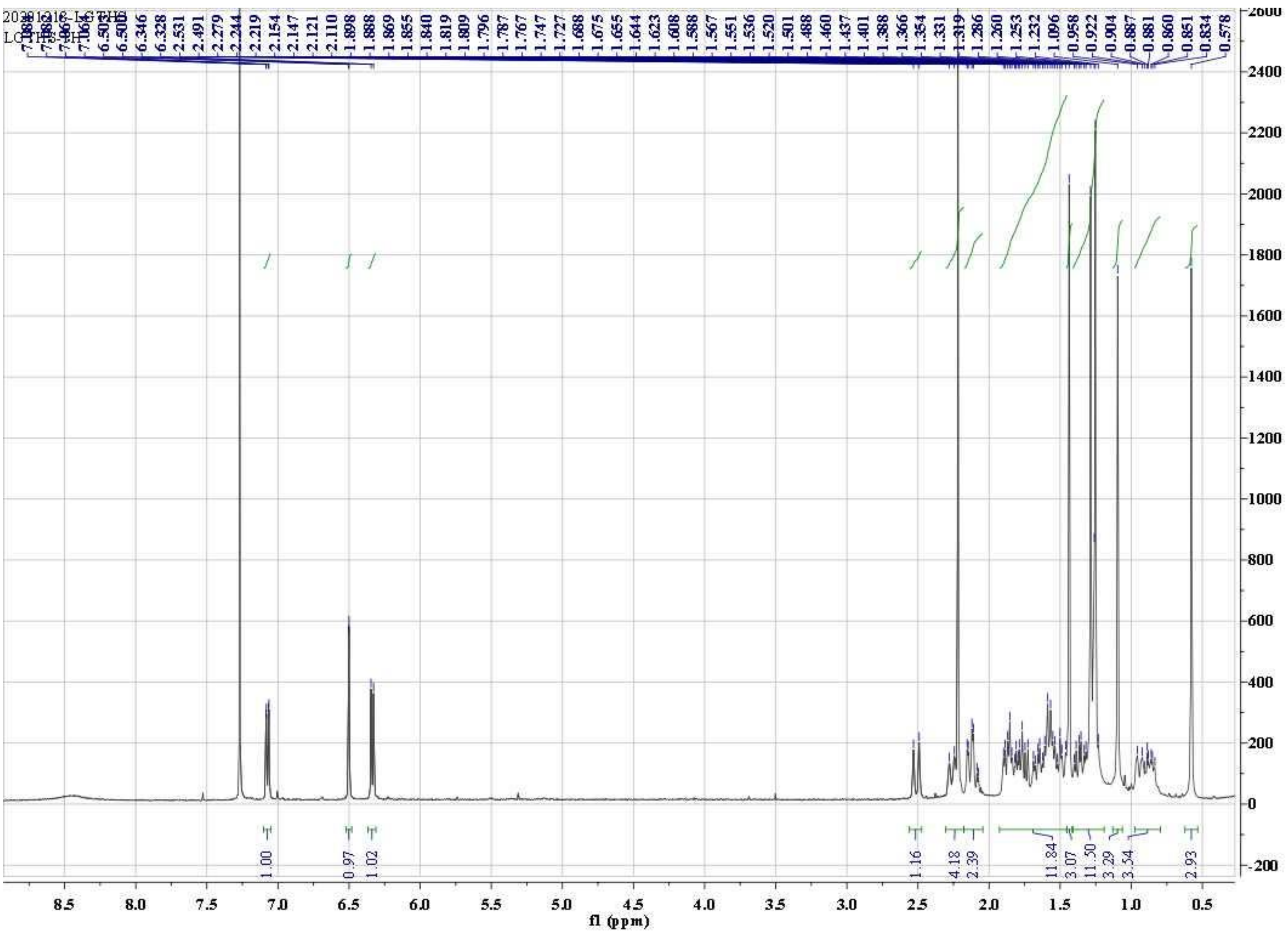

Figure S10 The ^{1}H NMR spectrum of celastrol (**1**) in $CDCl_3$ at 400 MHz.

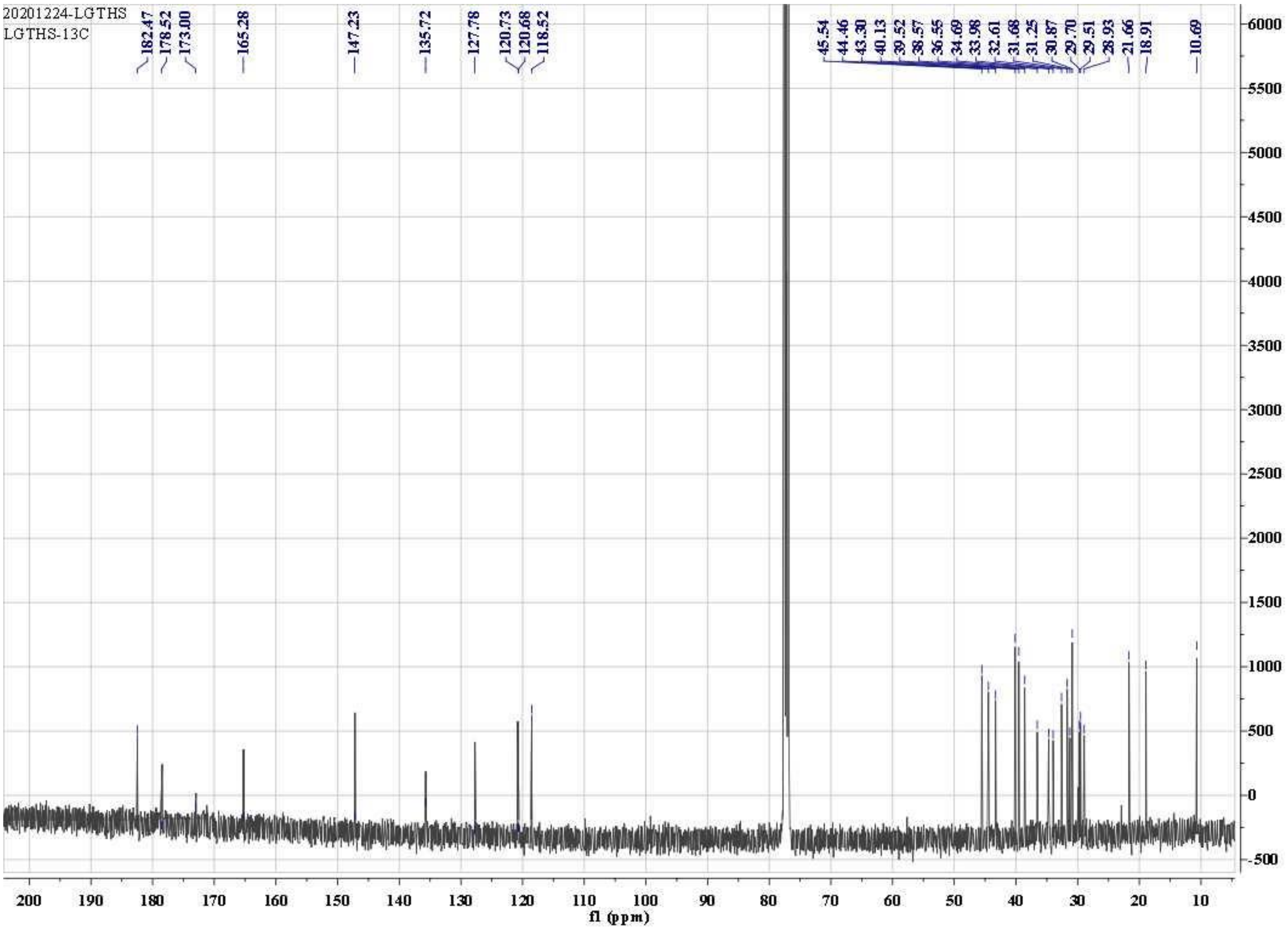

Figure S11 The ^{13}C NMR spectrum of celastrol (**1**) in $CDCl_3$ at 100 MHz.

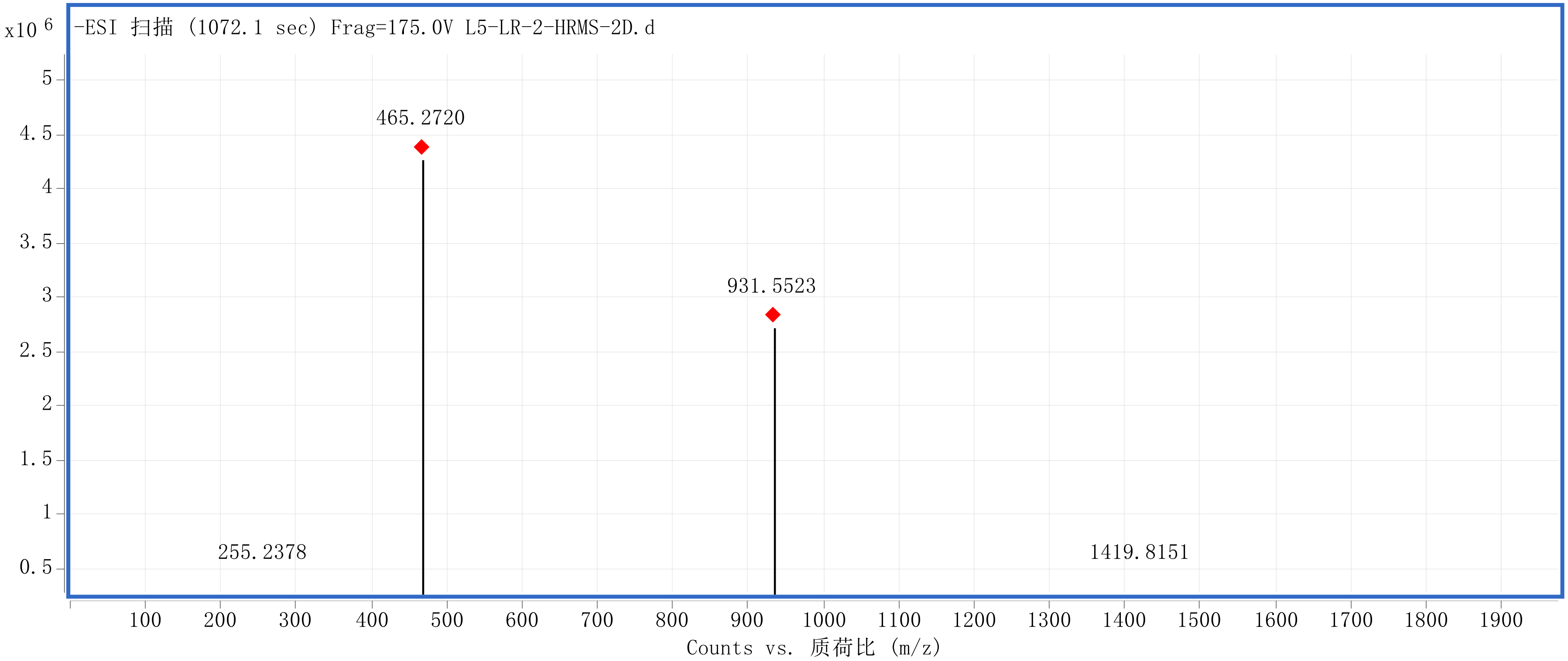

Figure S12 The (-)-HRESIMS spectrum of **2** (*m/z* 465.2720 $[M-H]^-$).

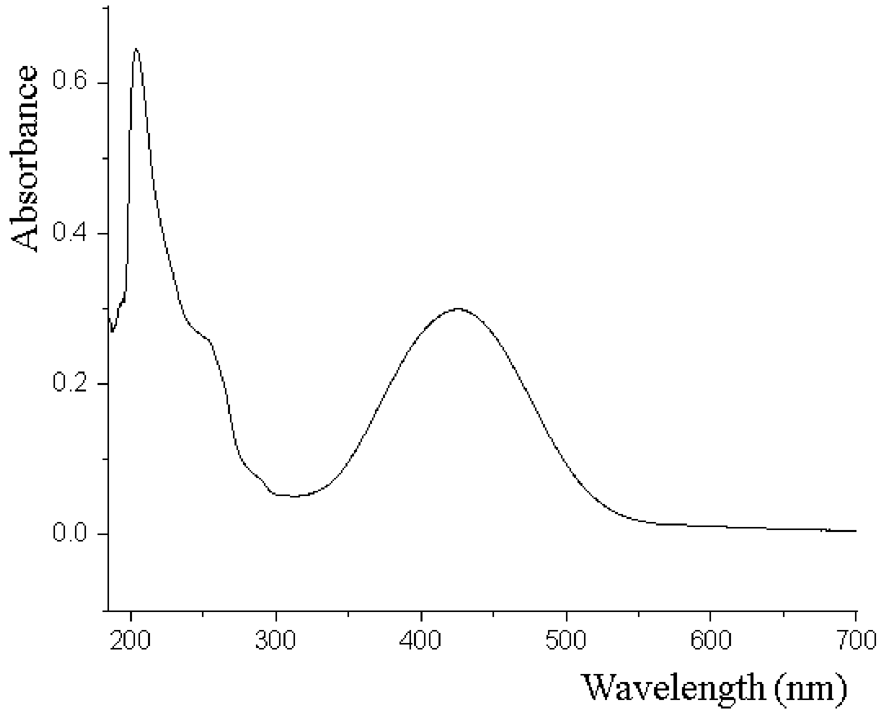

Figure S13 The UV spectrum of **2** in CH_3OH.

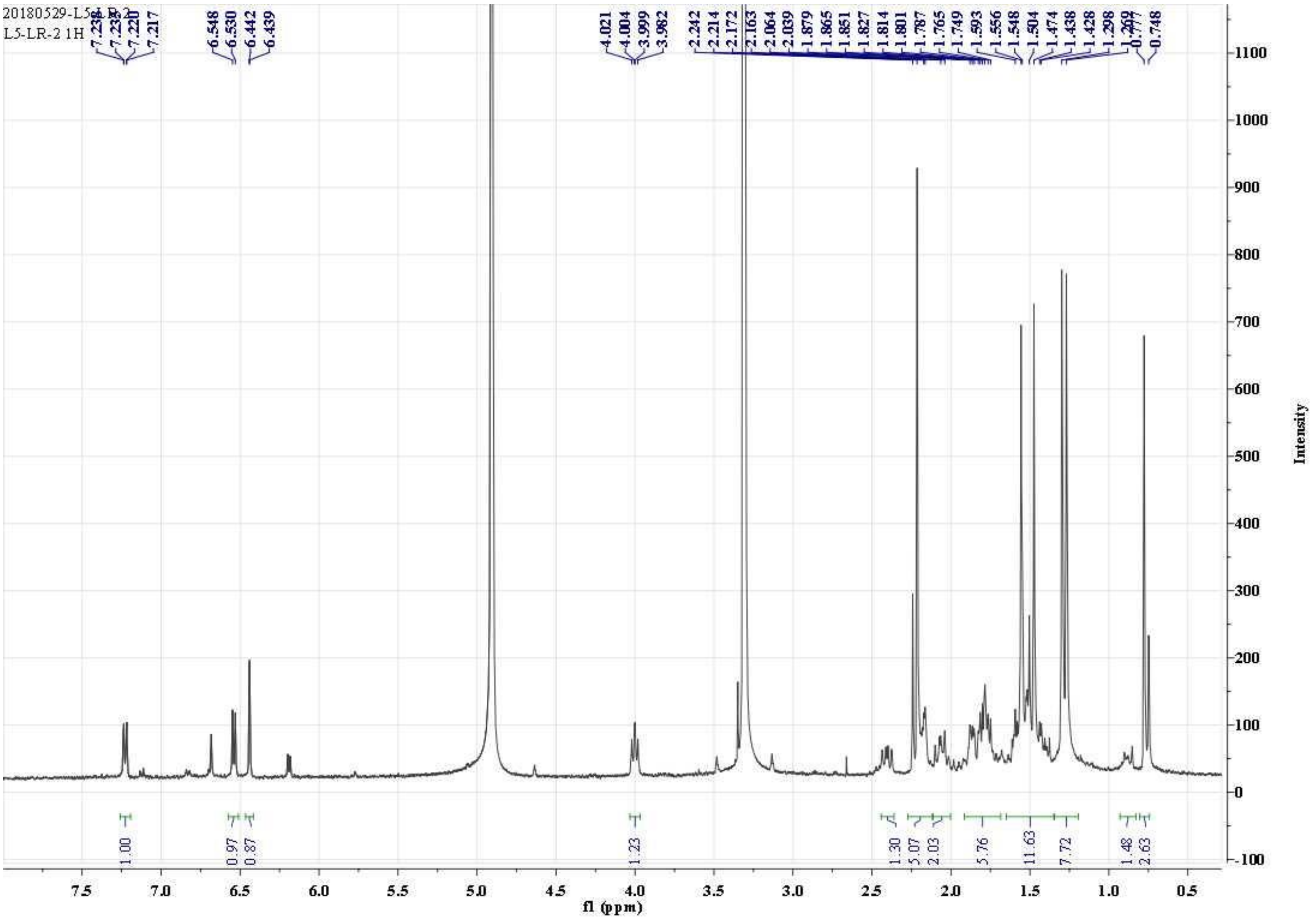

Figure S14 The ^{1}H NMR spectrum of **2** in CD_3OD at 400 MHz.

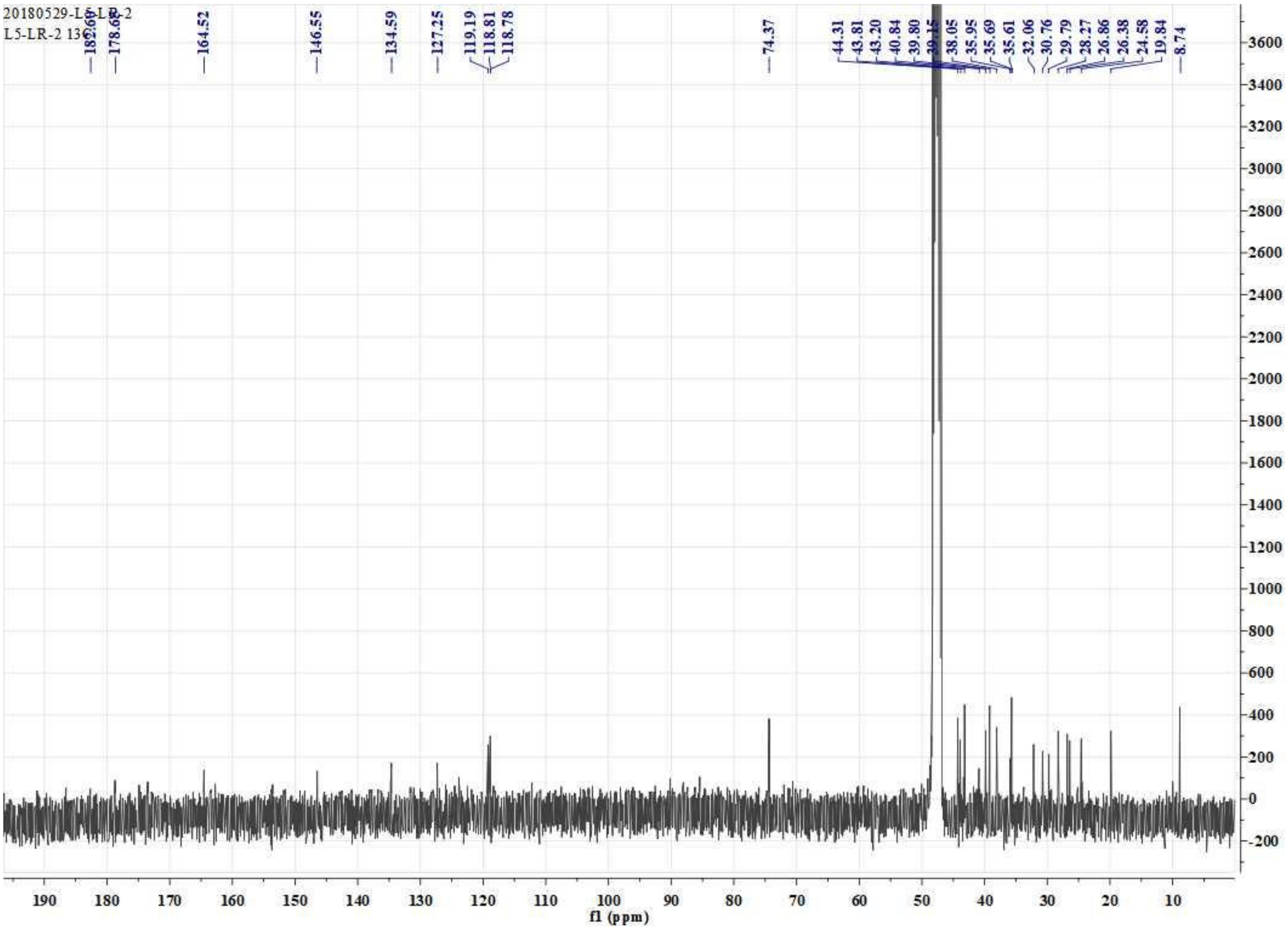

Figure S15 The ^{13}C NMR spectrum of **2** in CD_3OD at 100 MHz.

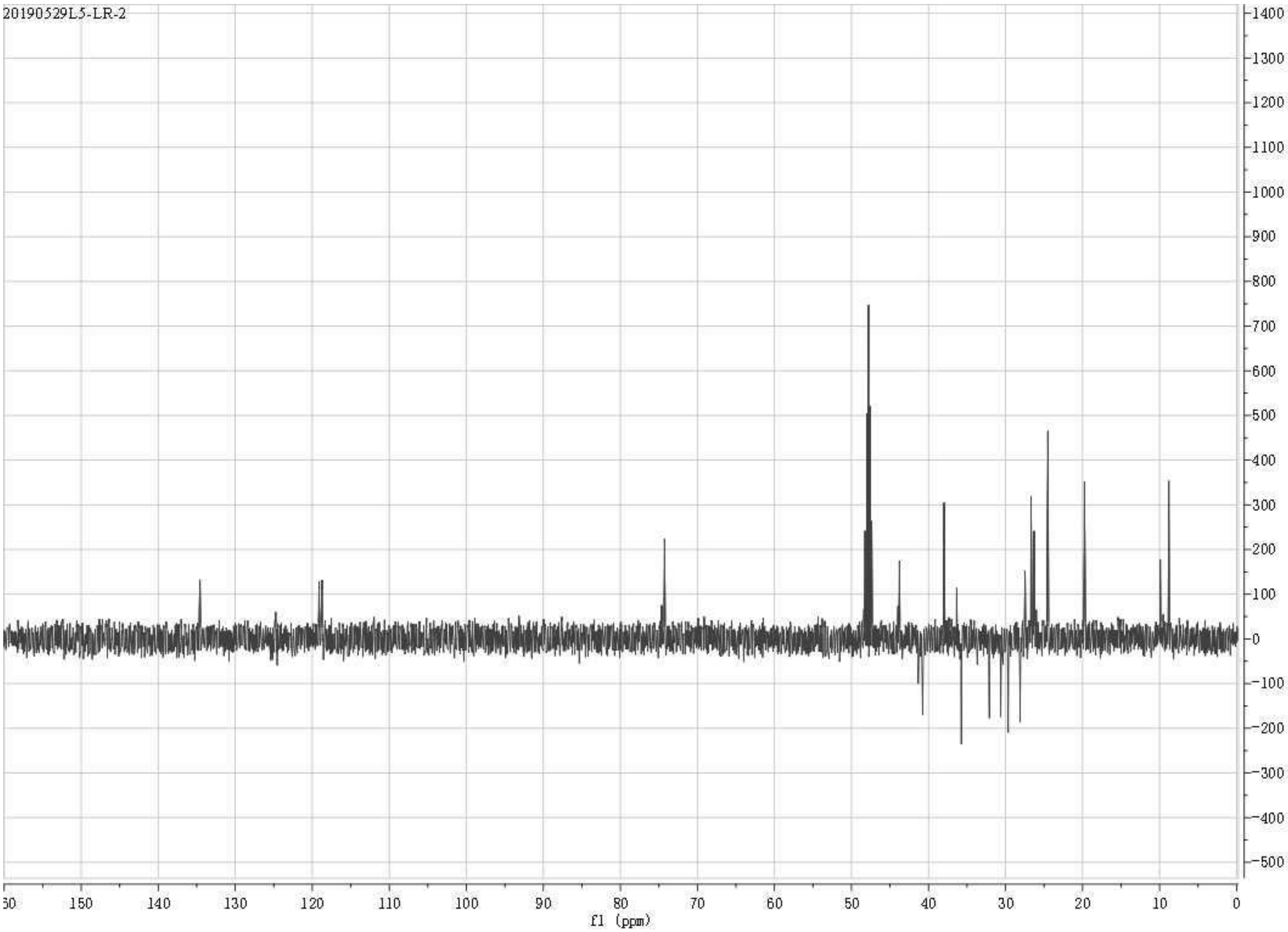

Figure S16 The DEPT spectrum of **2** in CD_3OD.

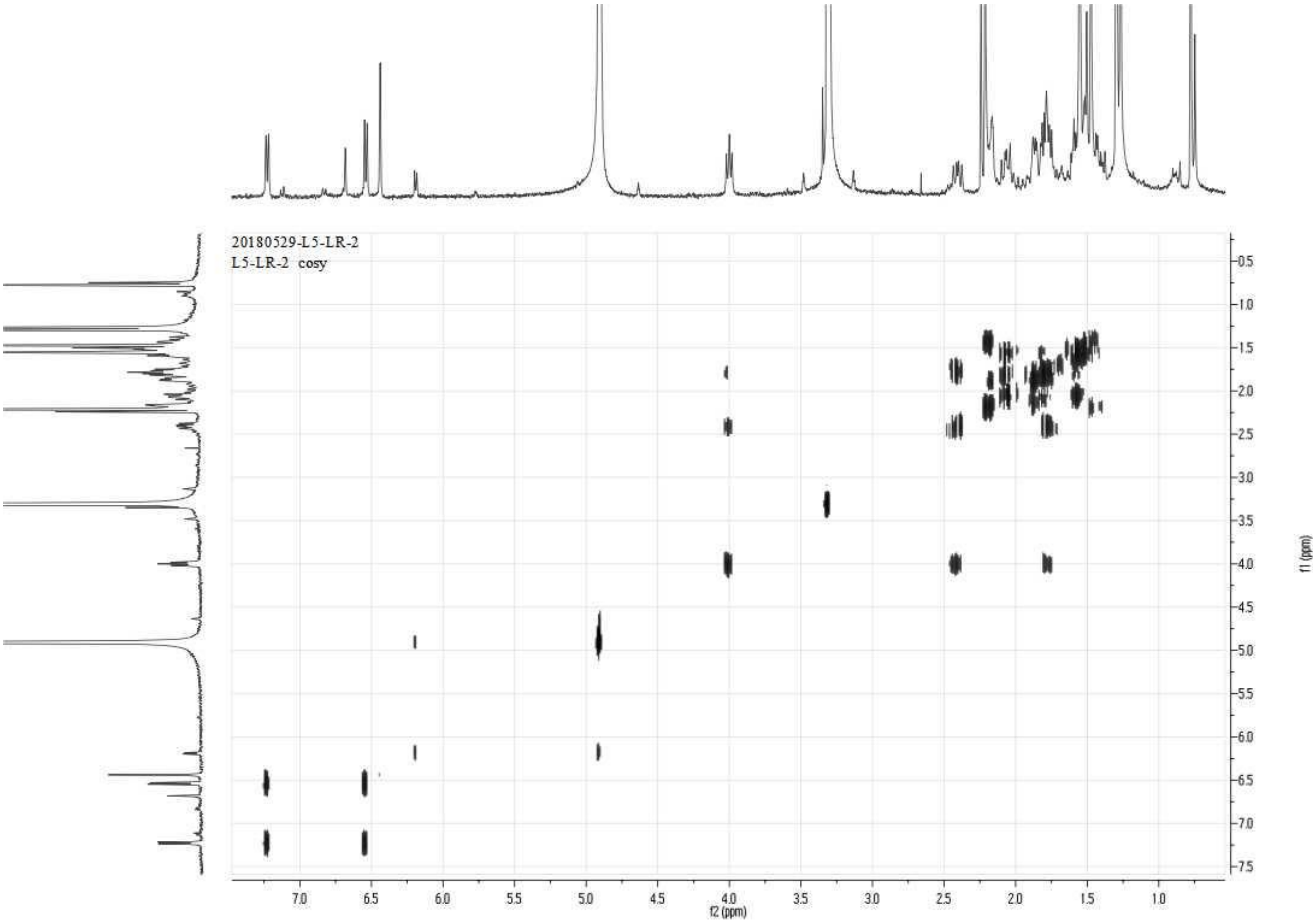

Figure S17 The ^{1}H-^{1}H COSY spectrum of **2** in CD_3OD.

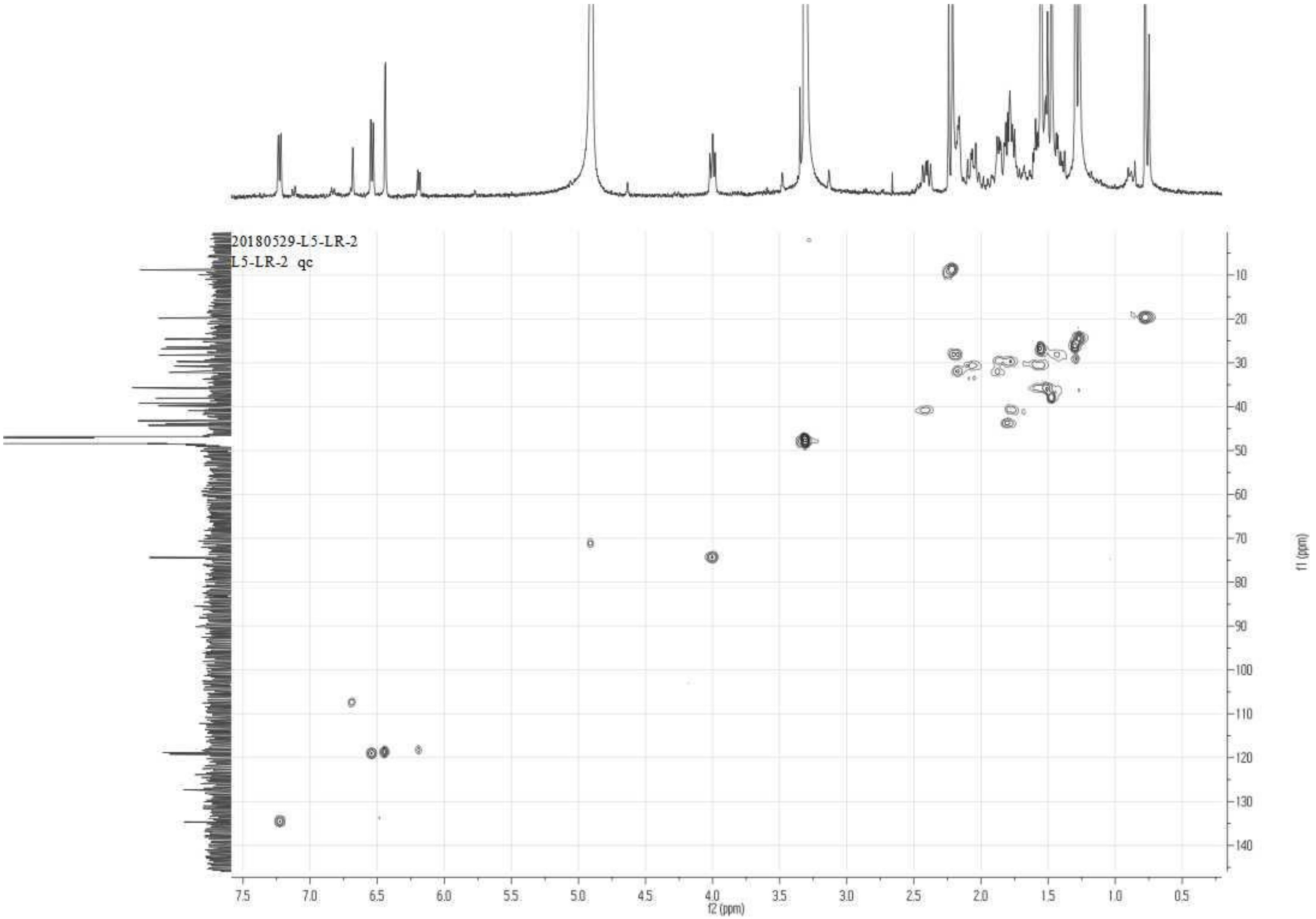

Figure S18 The HSQC spectrum of **2** in CD_3OD.

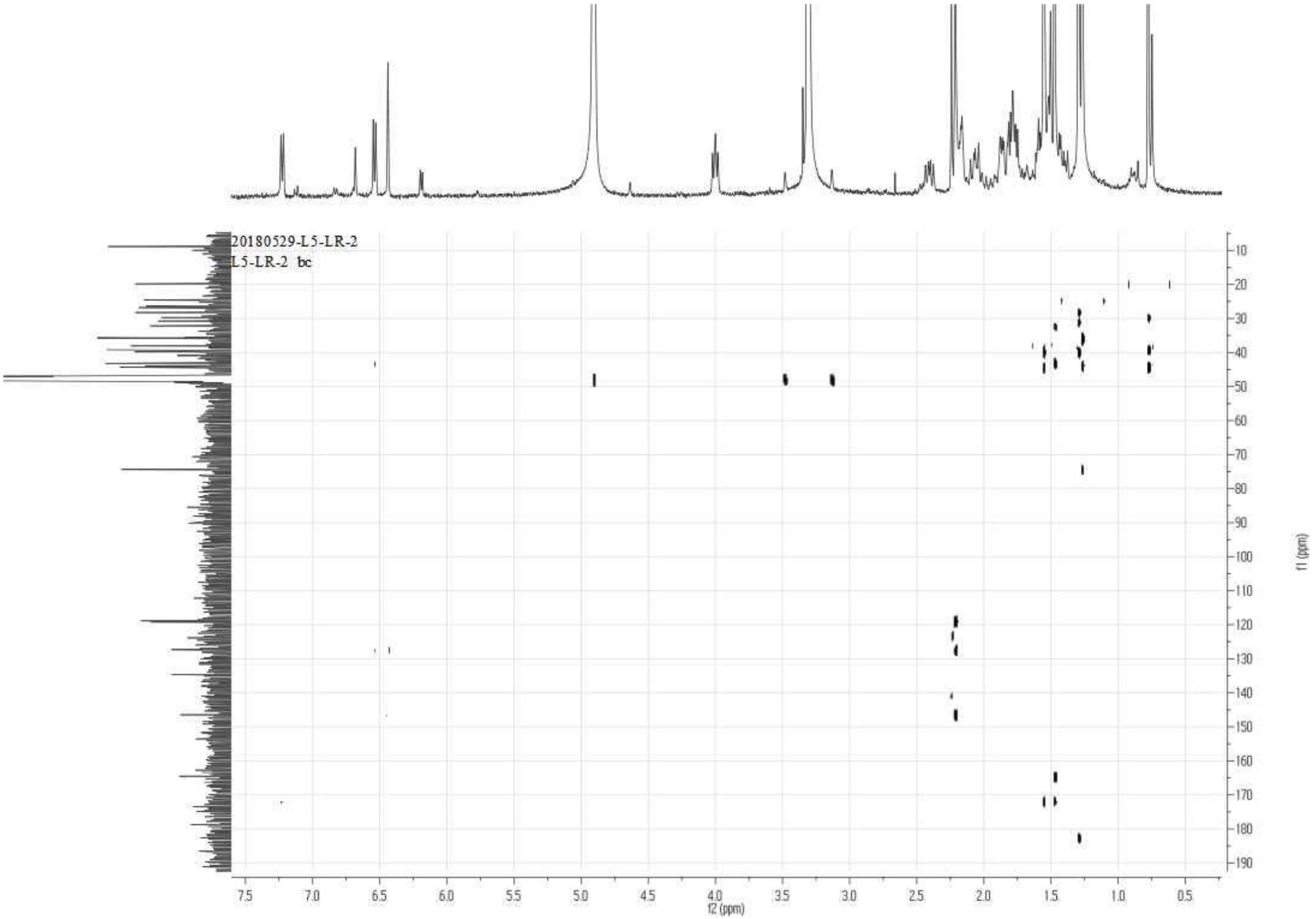

Figure S19 The HMBC spectrum of **2** in CD_3OD.

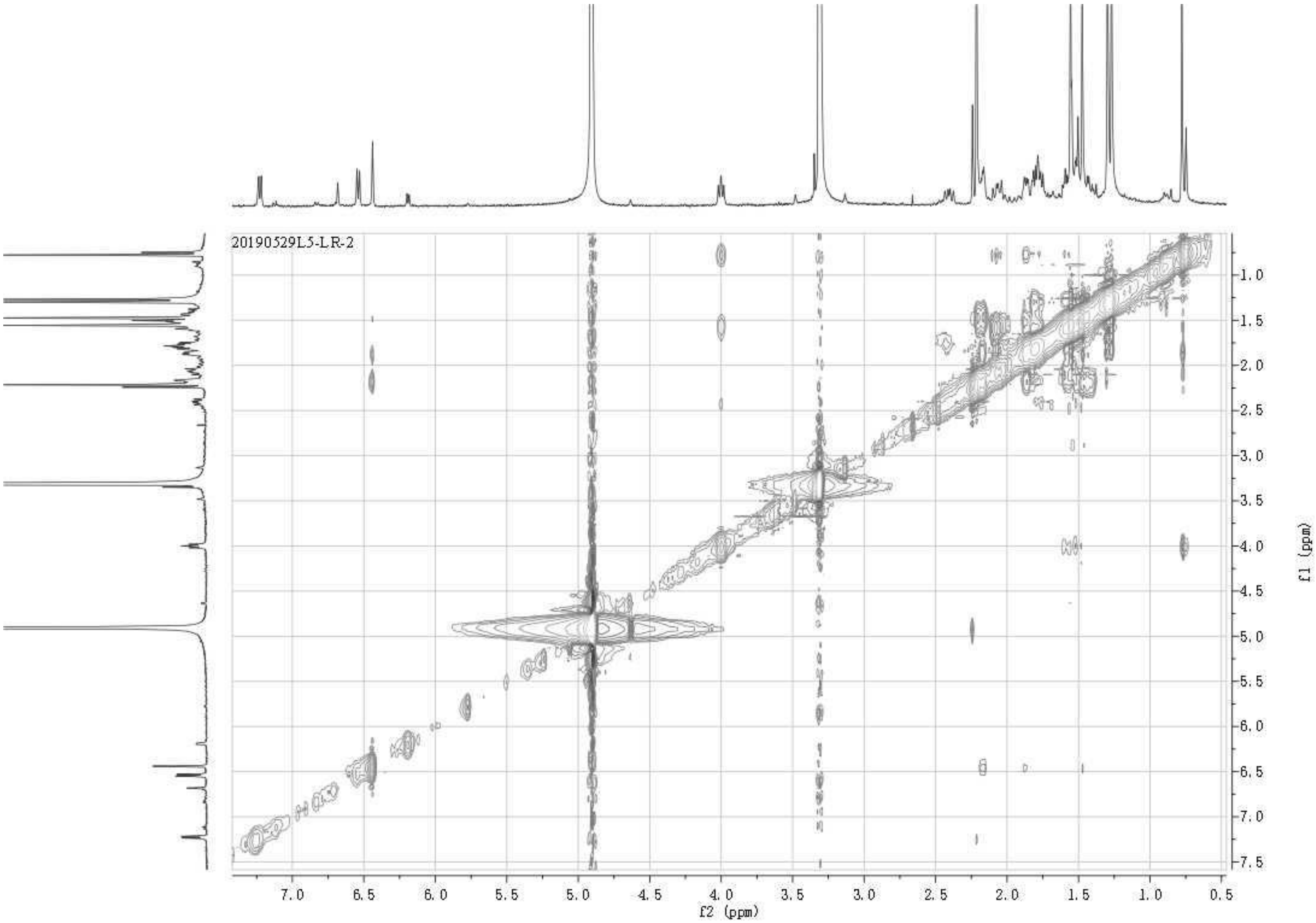

Figure S20 The NOESY spectrum of **2** in CD_3OD.

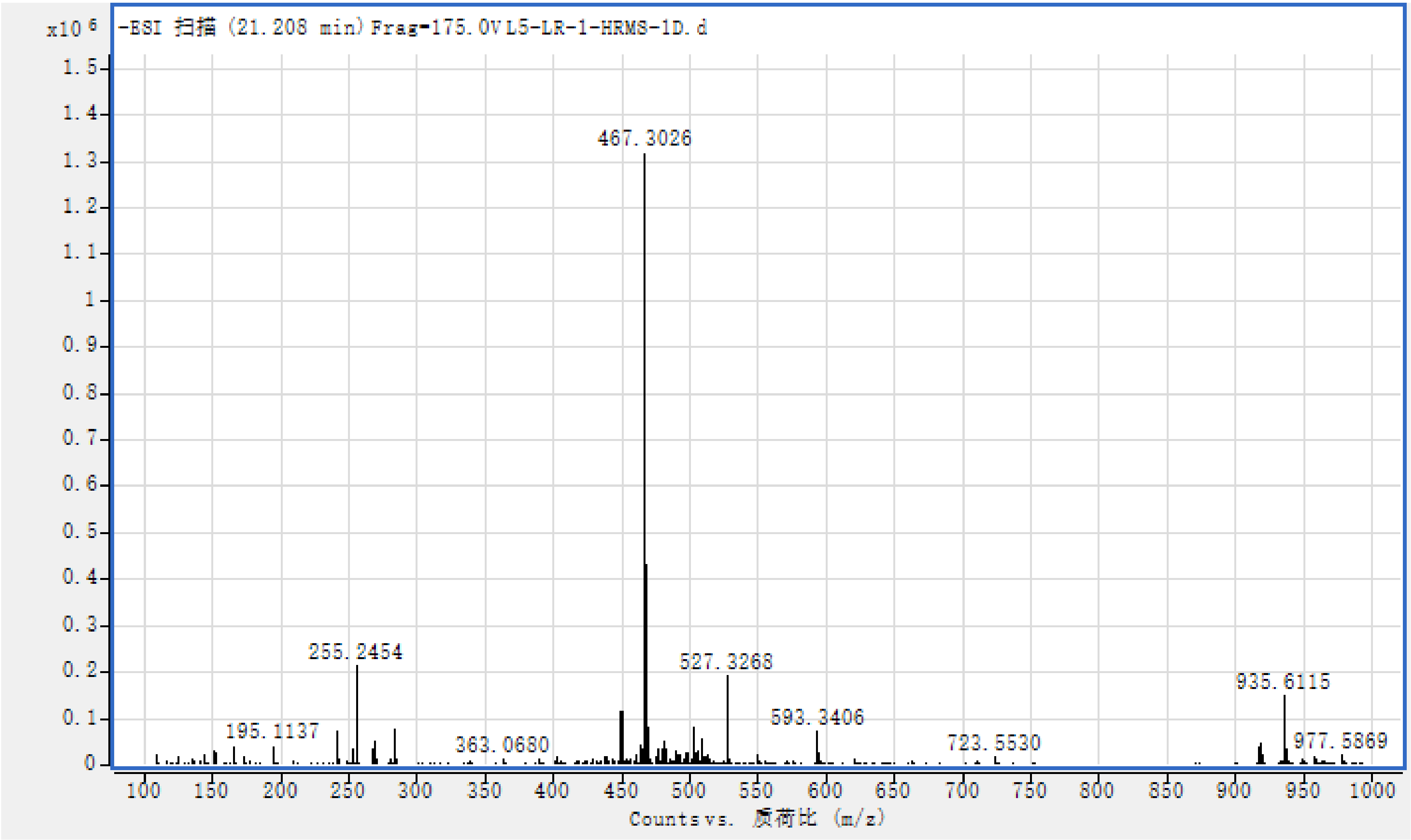

Figure S21 The HRESIMS spectrum of **3** at *m/z* 467.3026 [M - H]⁻.

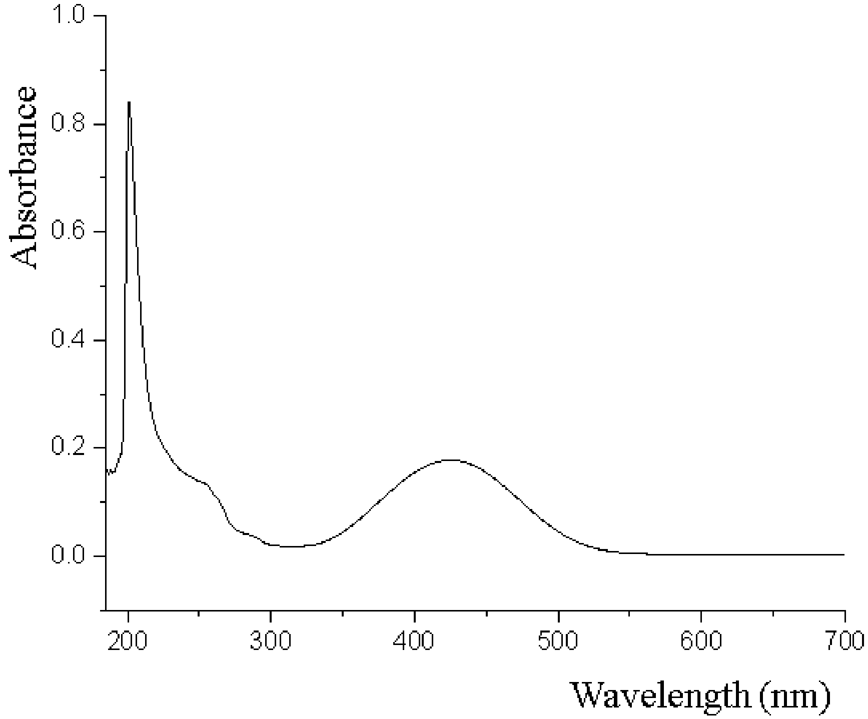

Figure S22 The UV spectrum of **3** in CH_3OH.

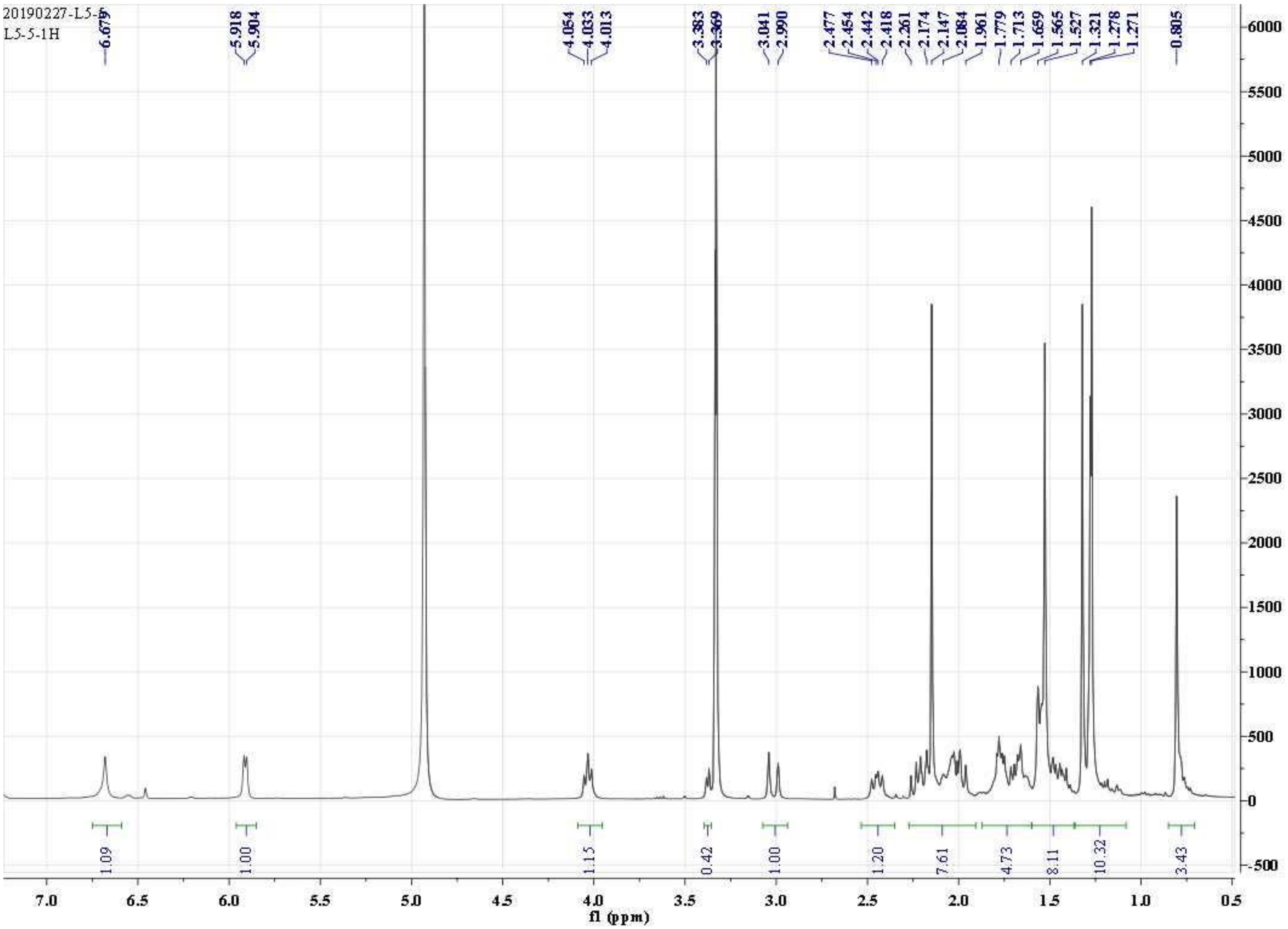

Figure S23 The ^{1}H NMR spectrum of **3** in CD_3OD at 400 MHz.

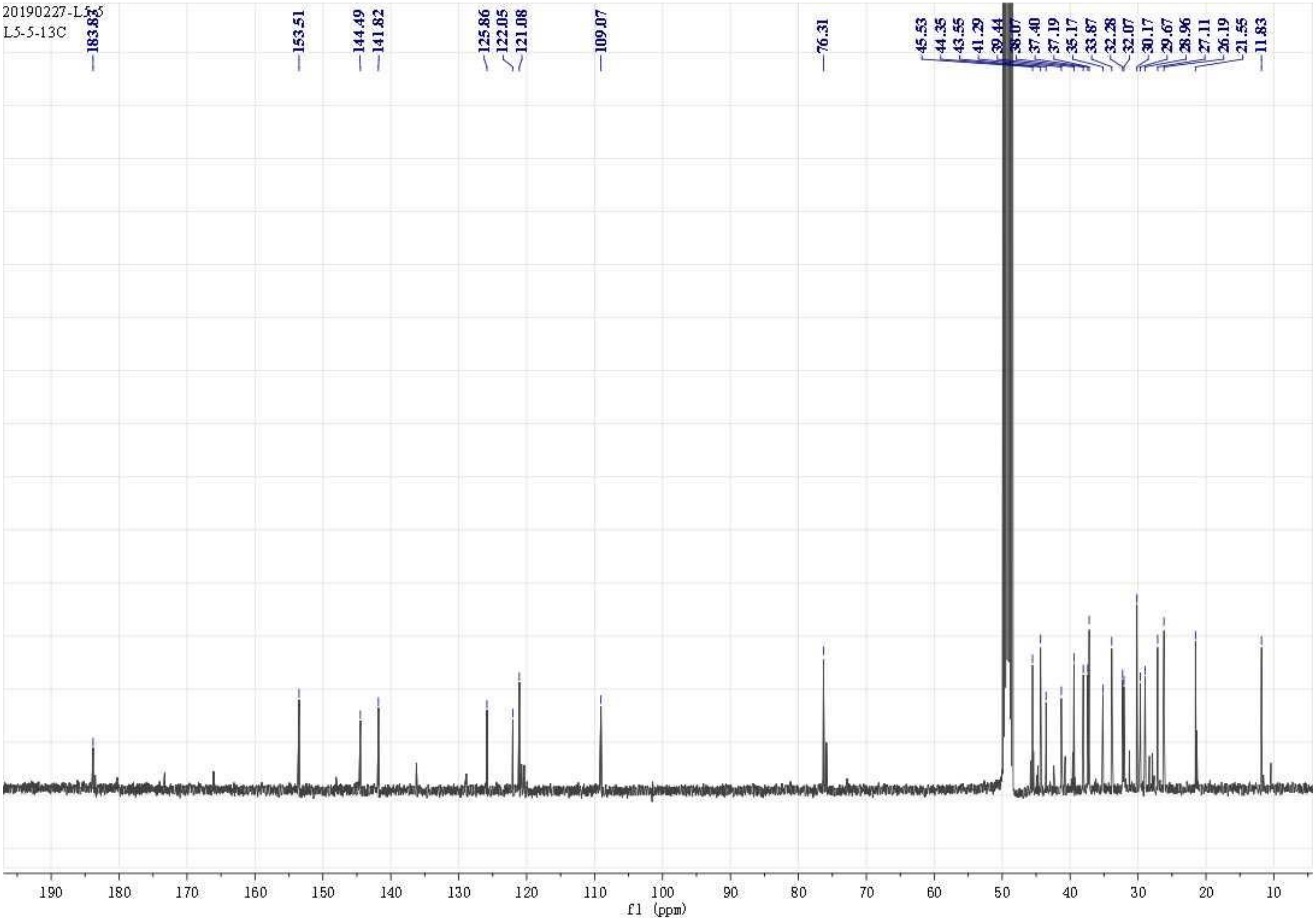

Figure S24 The ^{13}C NMR spectrum of **3** in CD_3OD at 100 MHz.

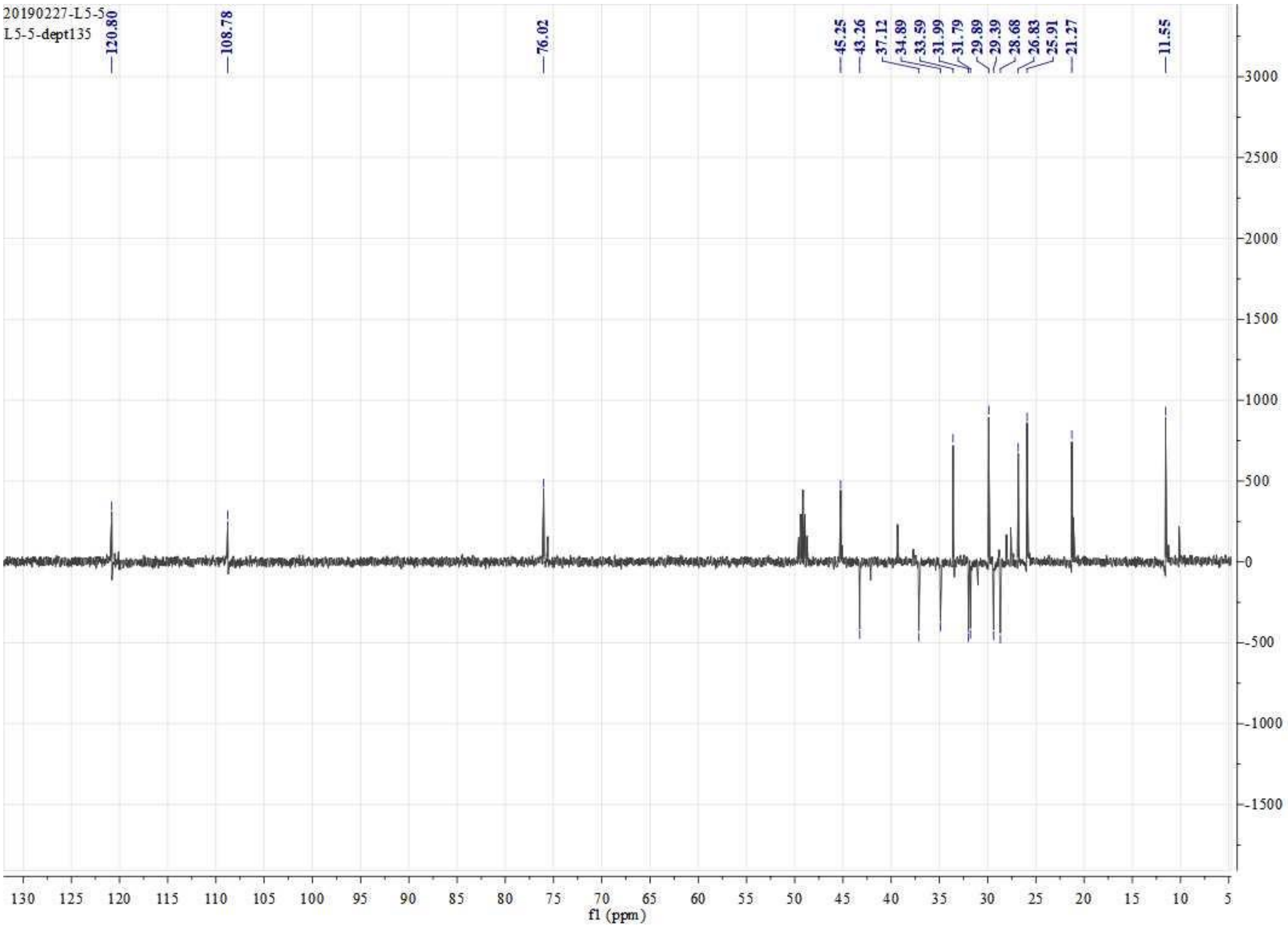

Figure S25 The DEPT spectrum of **3** in CD_3OD.

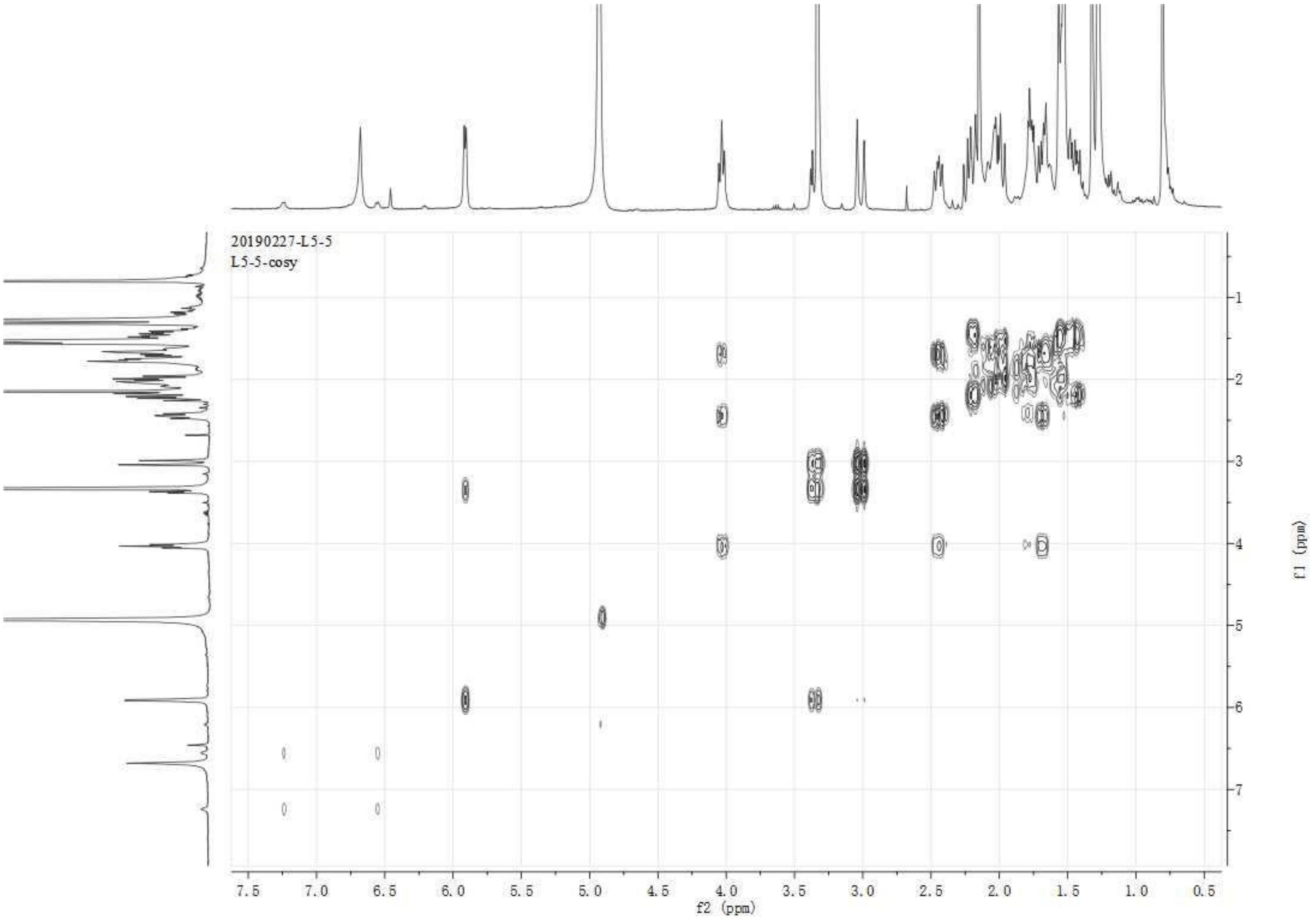

Figure S26 The ^{1}H-^{1}H COSY spectrum of **3** in CD_3OD.

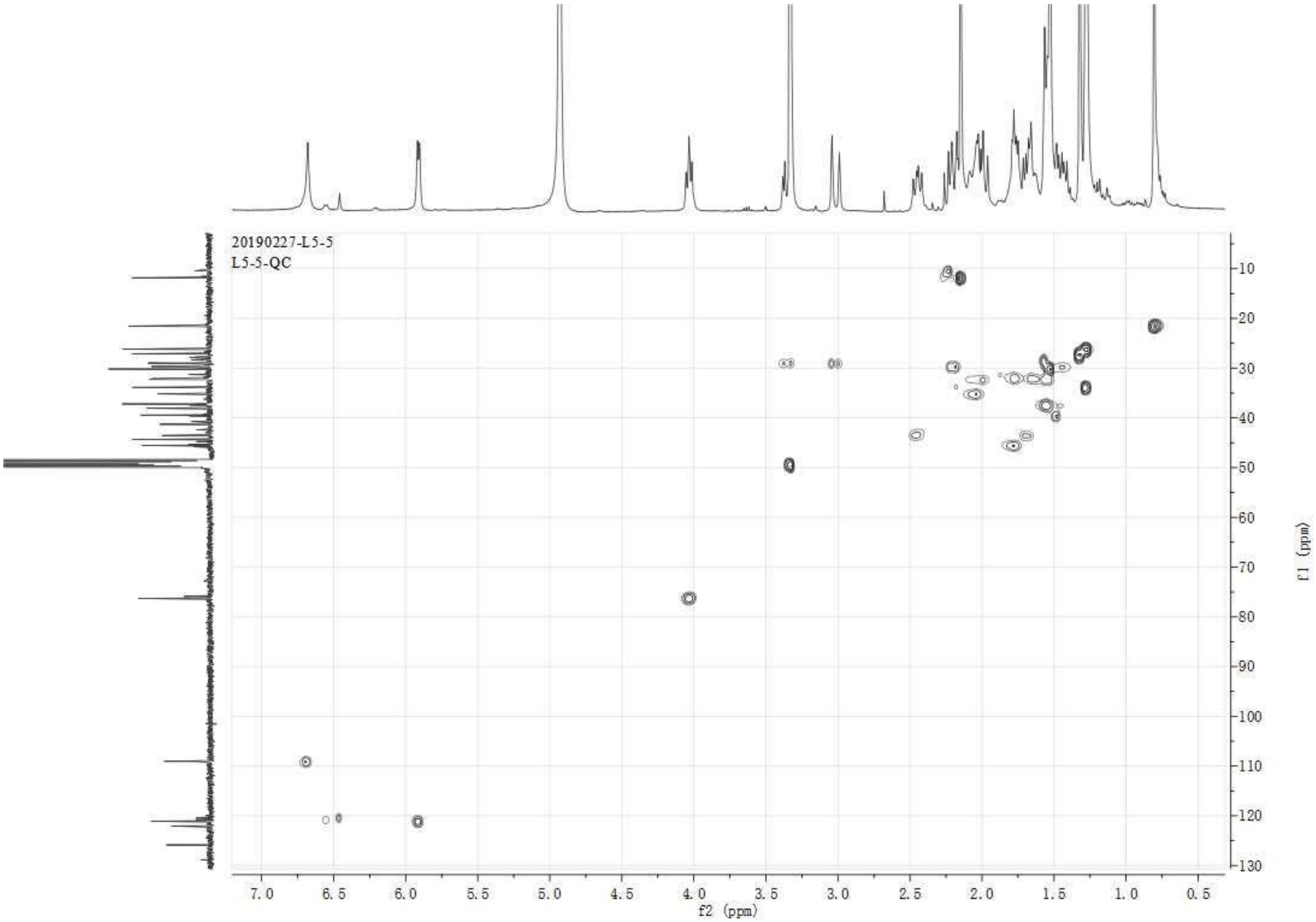

Figure S27 The HSQC spectrum of **3** in CD_3OD.

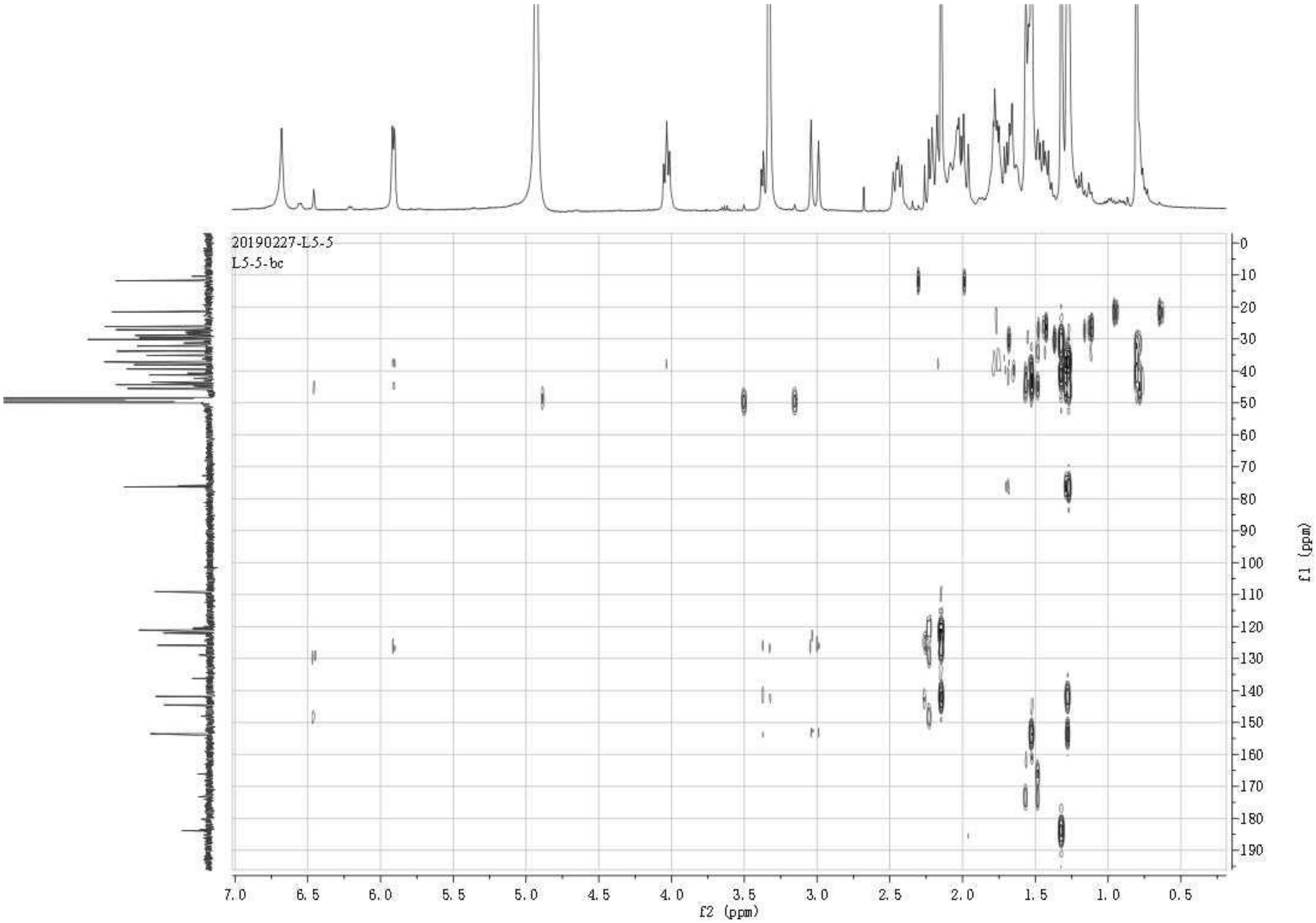

Figure S28 The HMBC spectrum of **3** in CD_3OD.

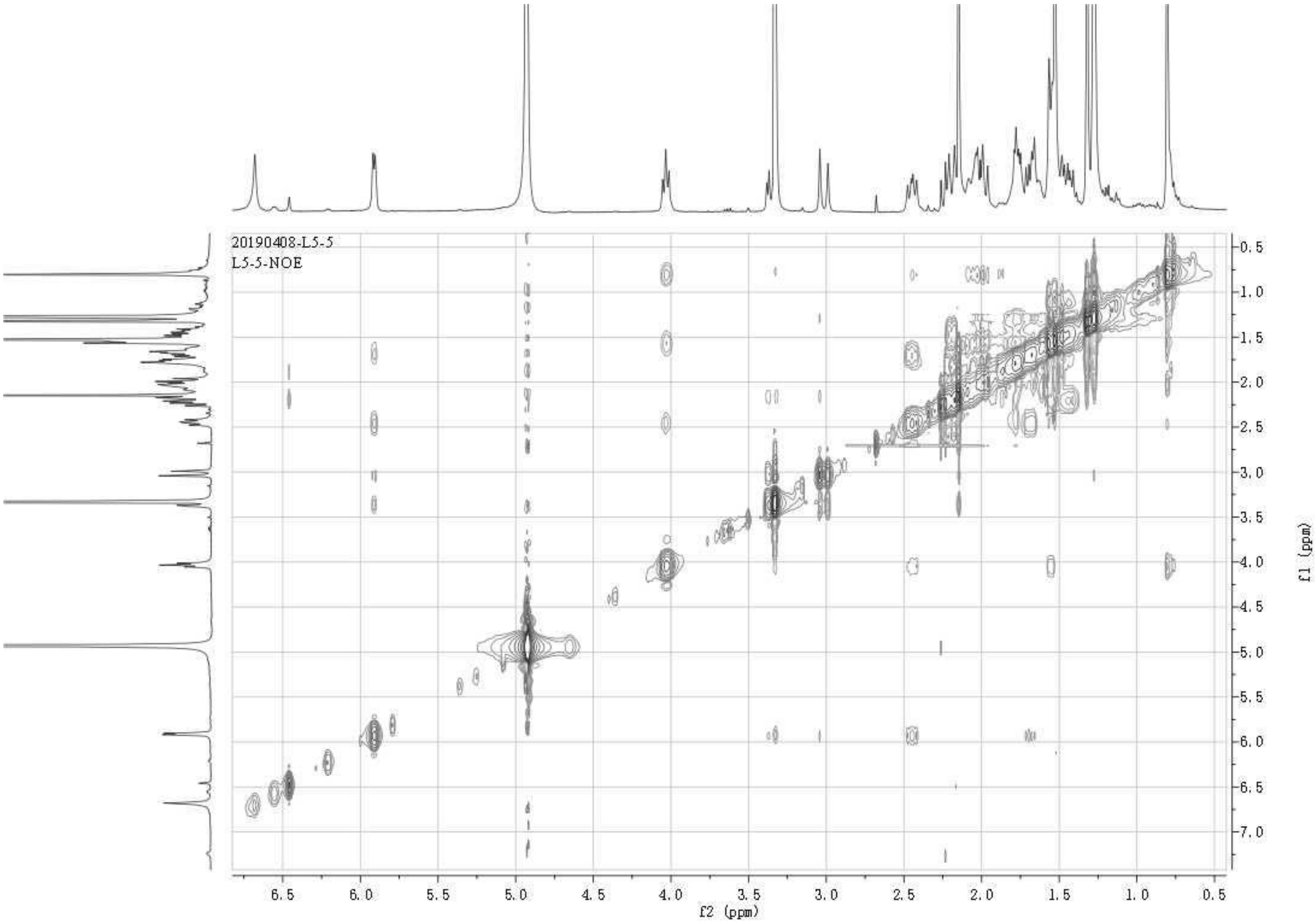

Figure S29 The NOESY spectrum of **3** in CD_3OD.

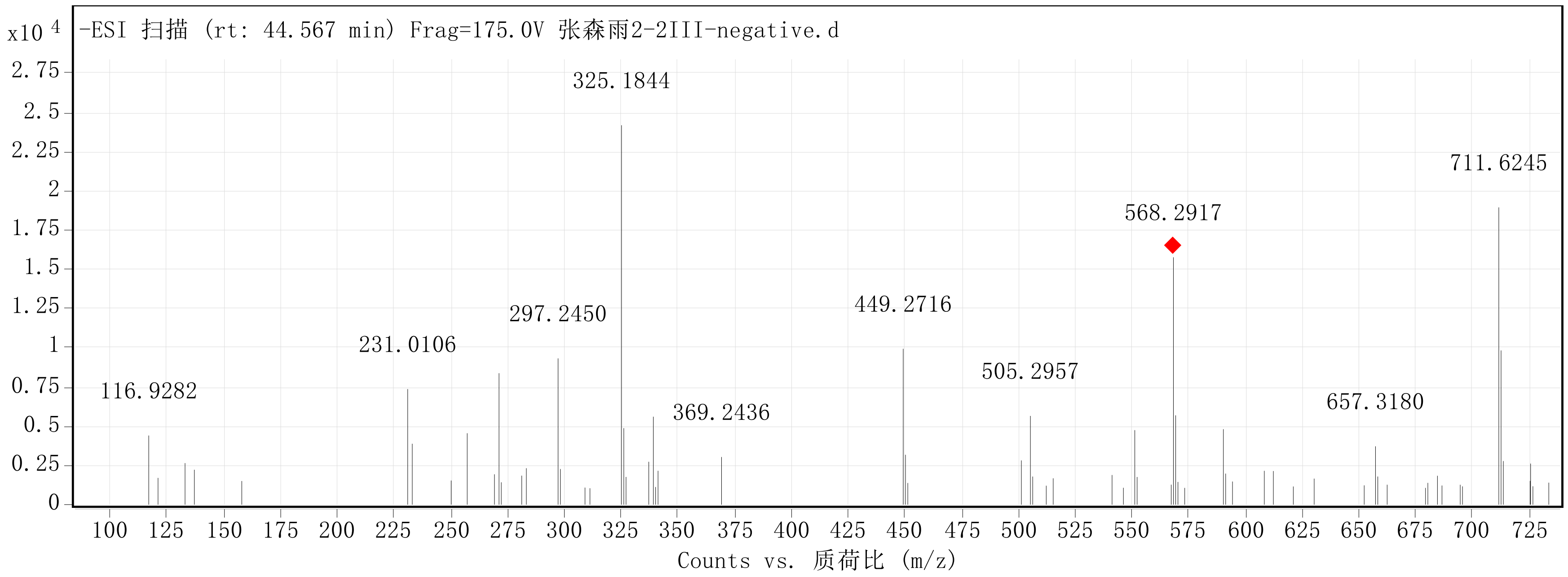

Figure S30 The HRESIMS spectrum of **4** at *m/z* 711.6245 [M - H]$^-$.

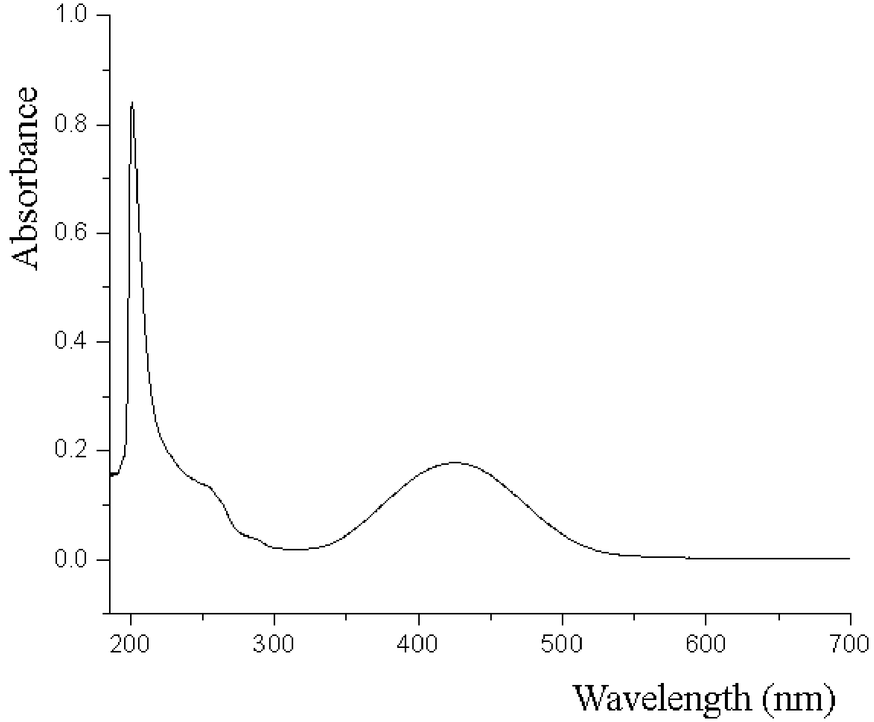

Figure S31 The UV spectrum of **4** in CH_3Cl.

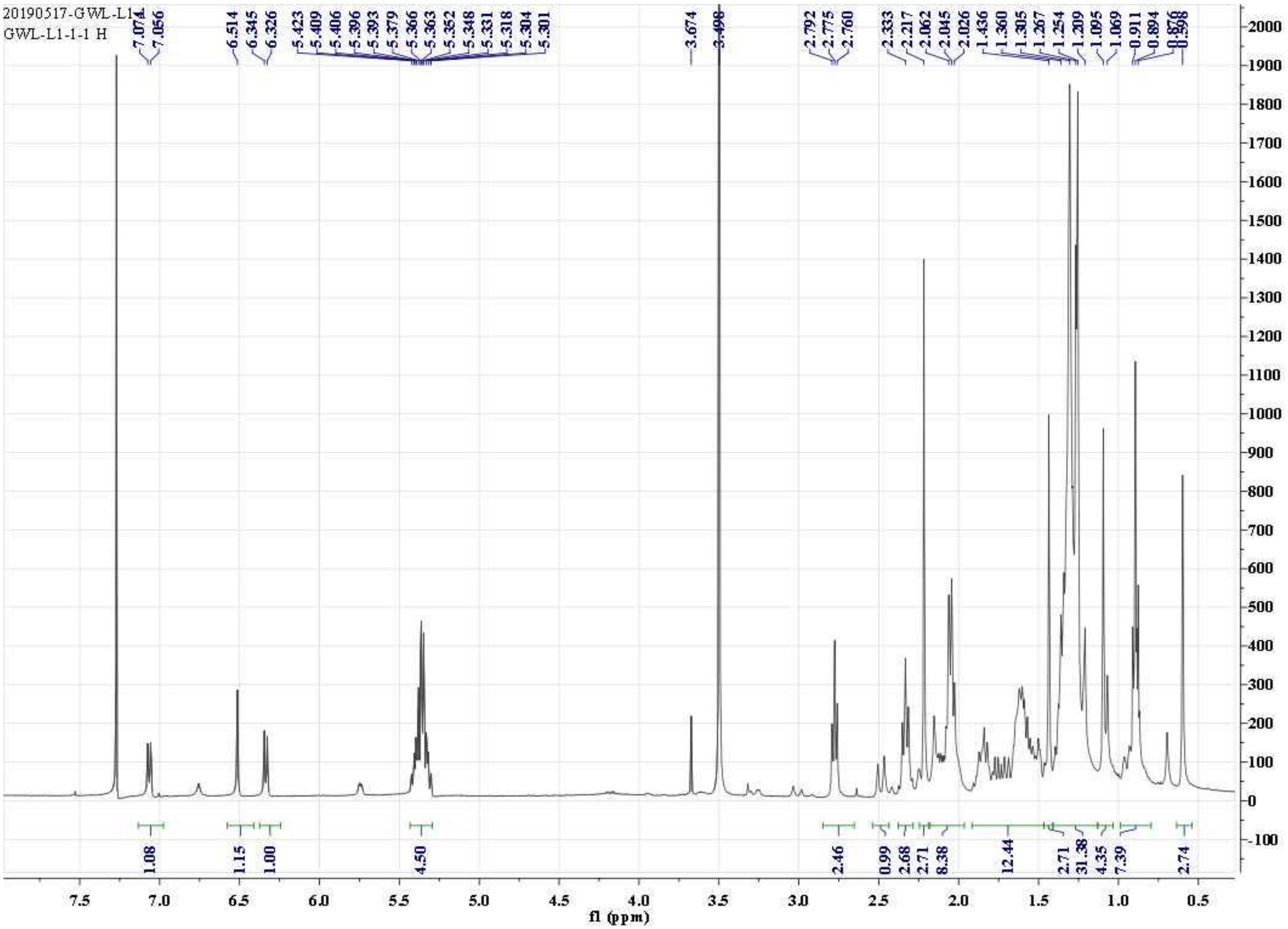

Figure S32 The ^{1}H NMR spectrum of **4** in $CDCl_3$ at 400 MHz.

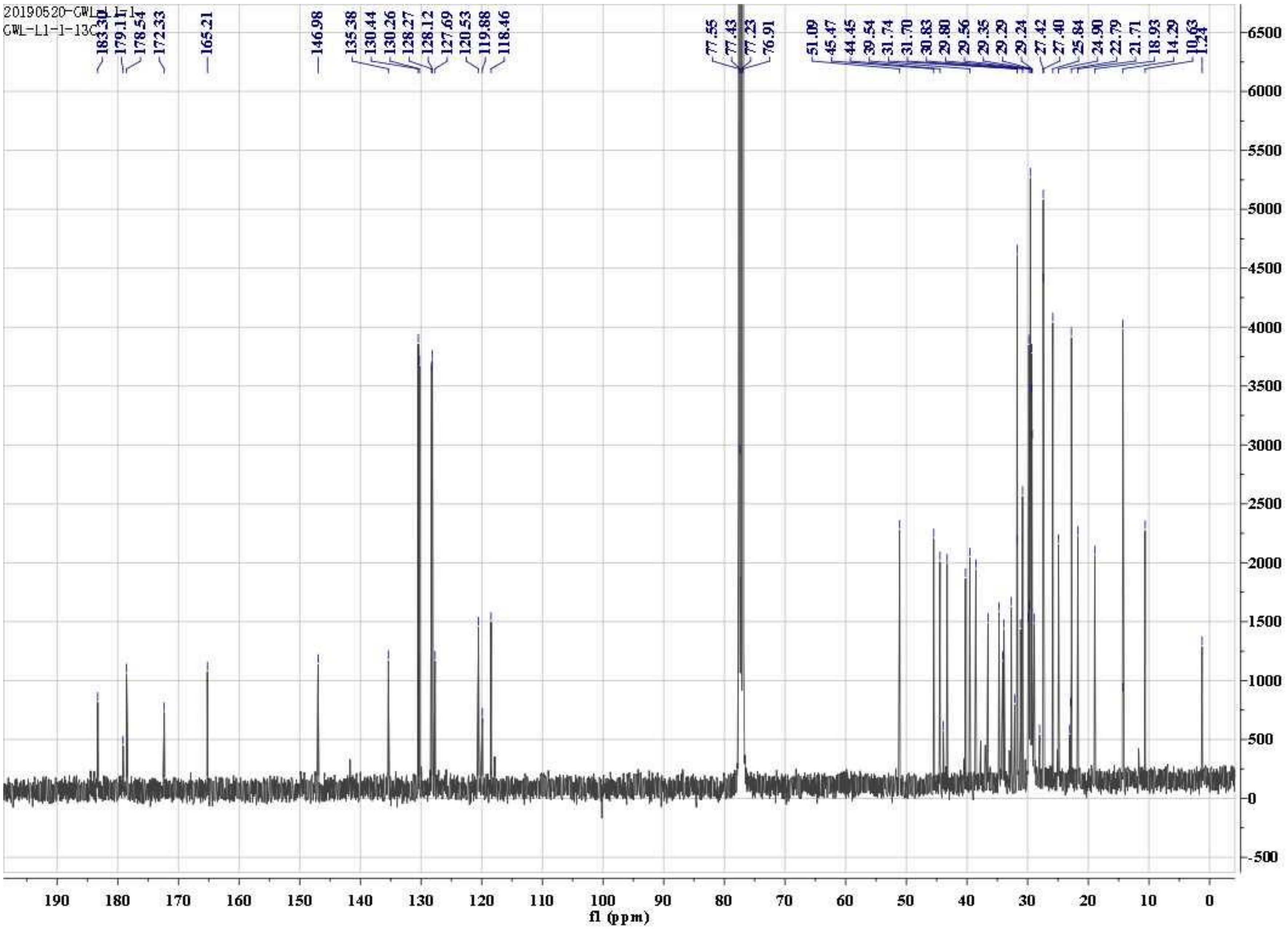

Figure S33 The ^{13}C NMR spectrum of **4** in $CDCl_3$ at 100 MHz.

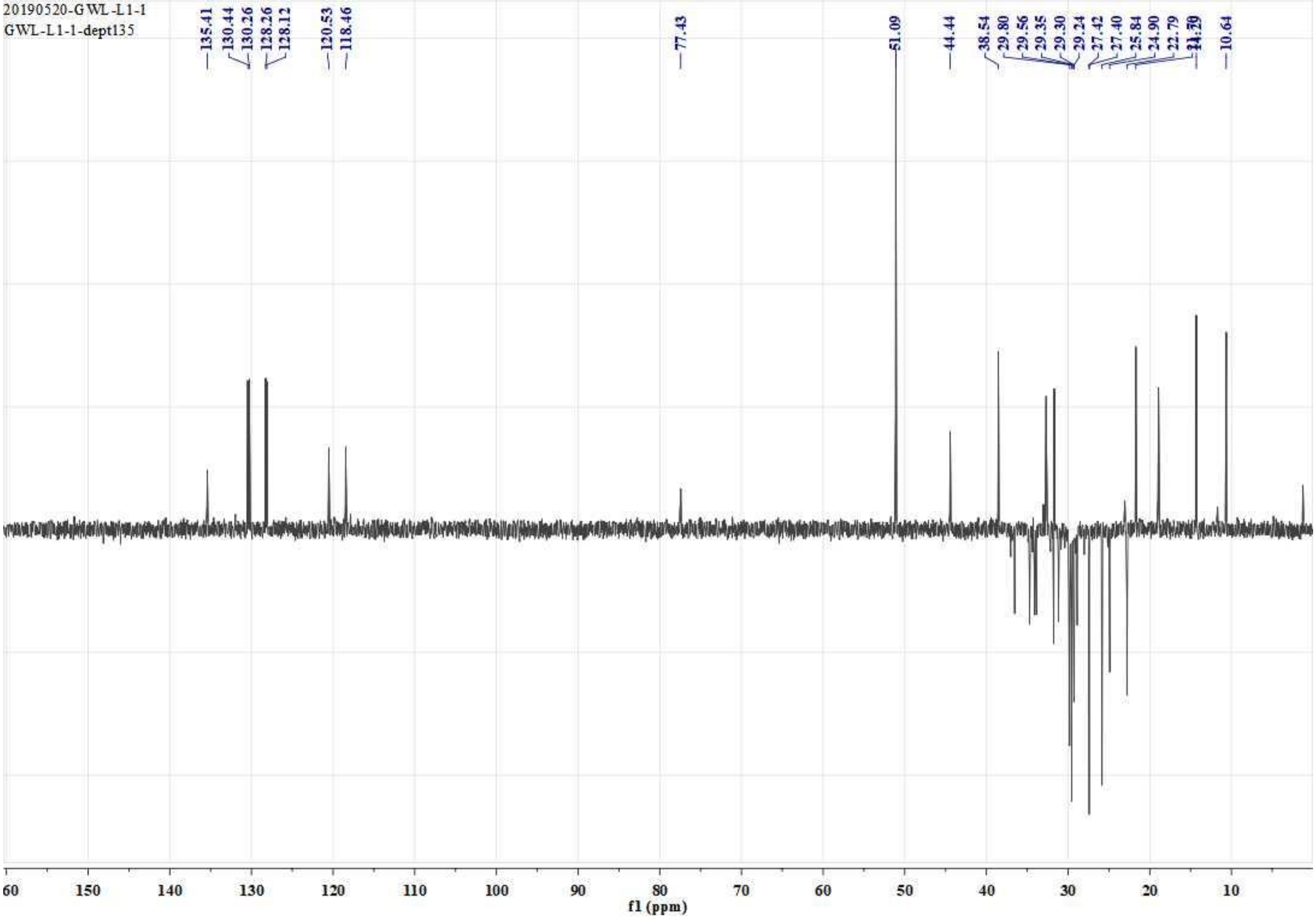

Figure S34 The DEPT spectrum of **4** in $CDCl_3$.

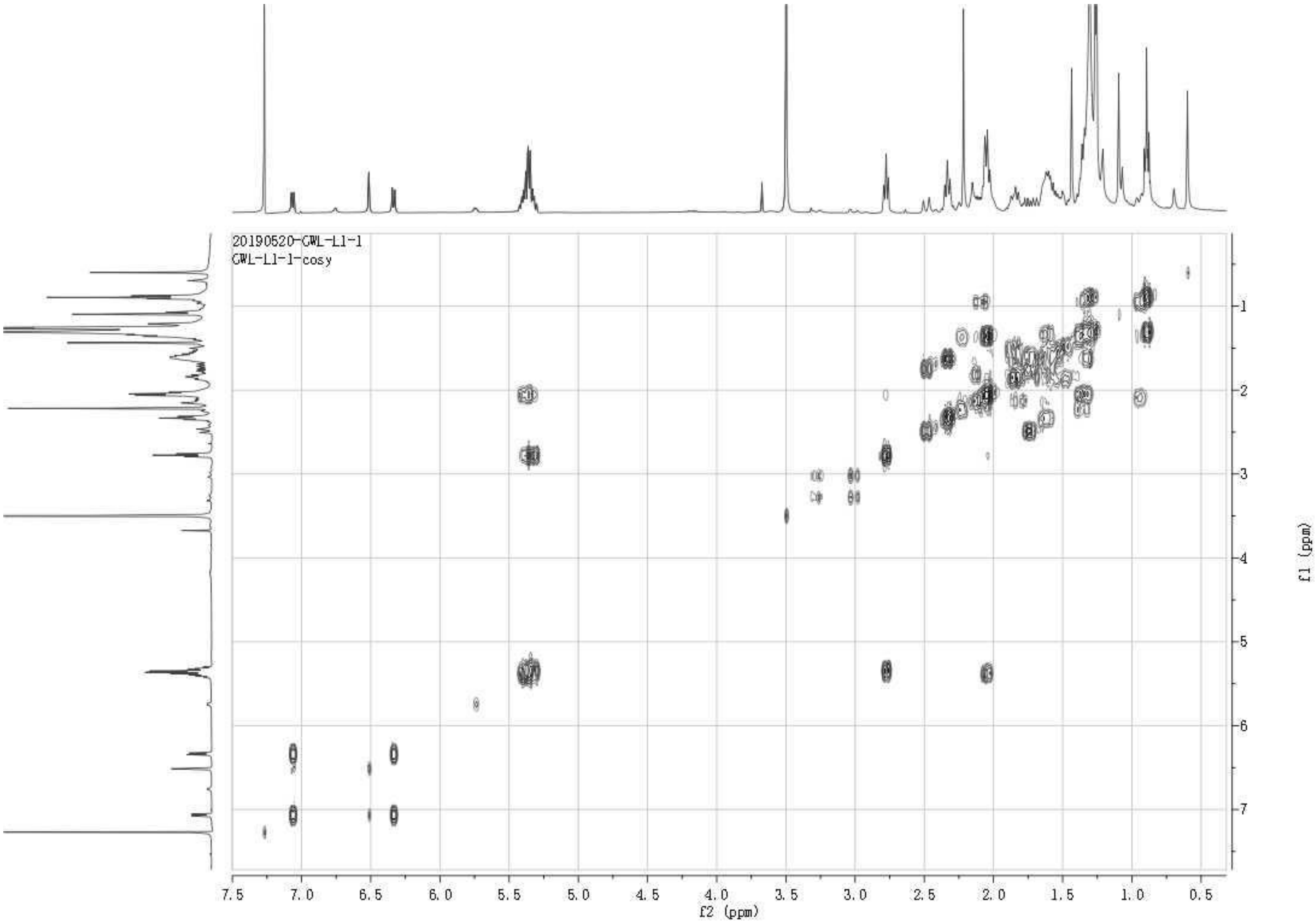

Figure S35 The ^{1}H-^{1}H COSY spectrum of **4** in $CDCl_3$.

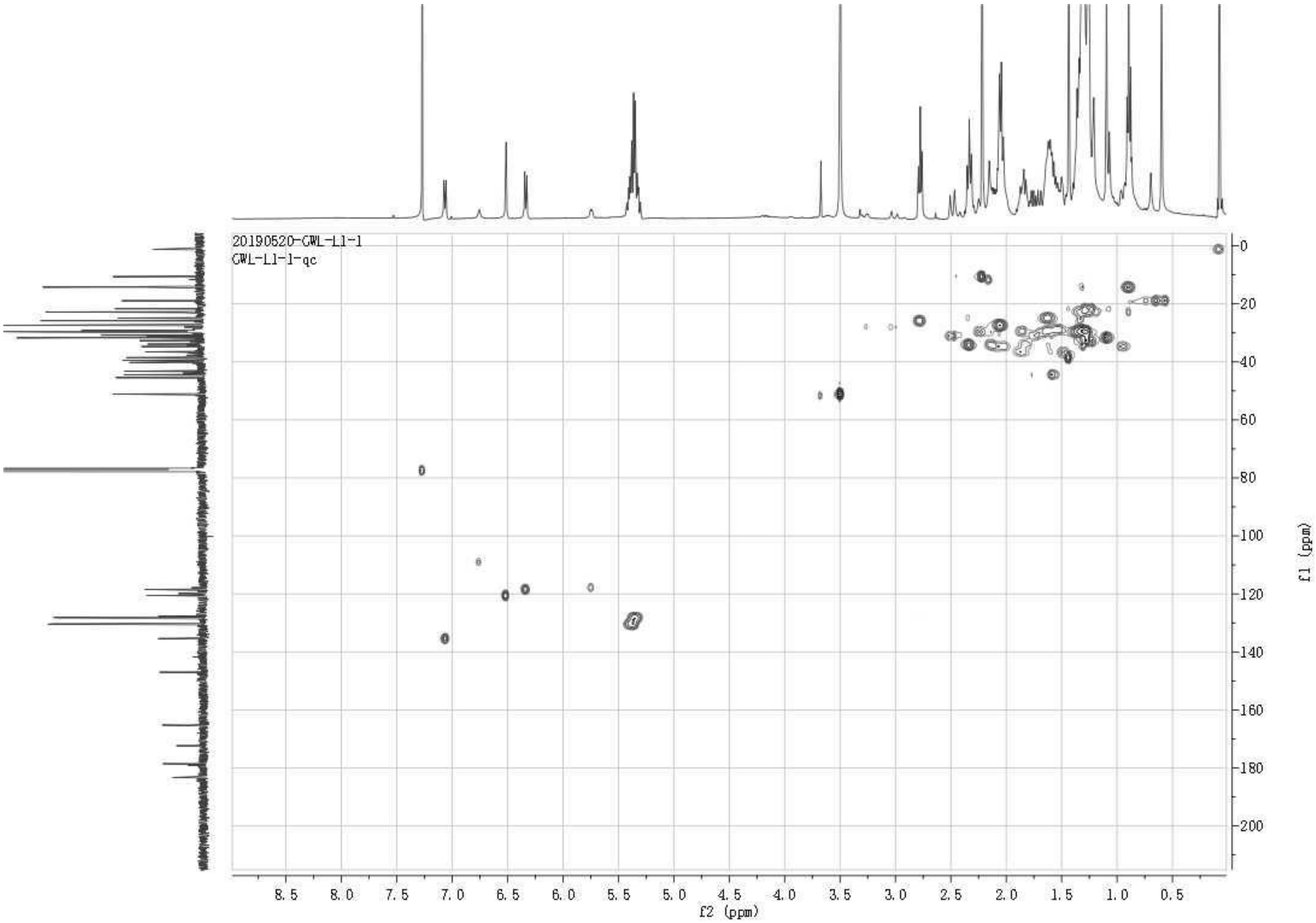

Figure S36 The HSQC spectrum of **4** in $CDCl_3$.

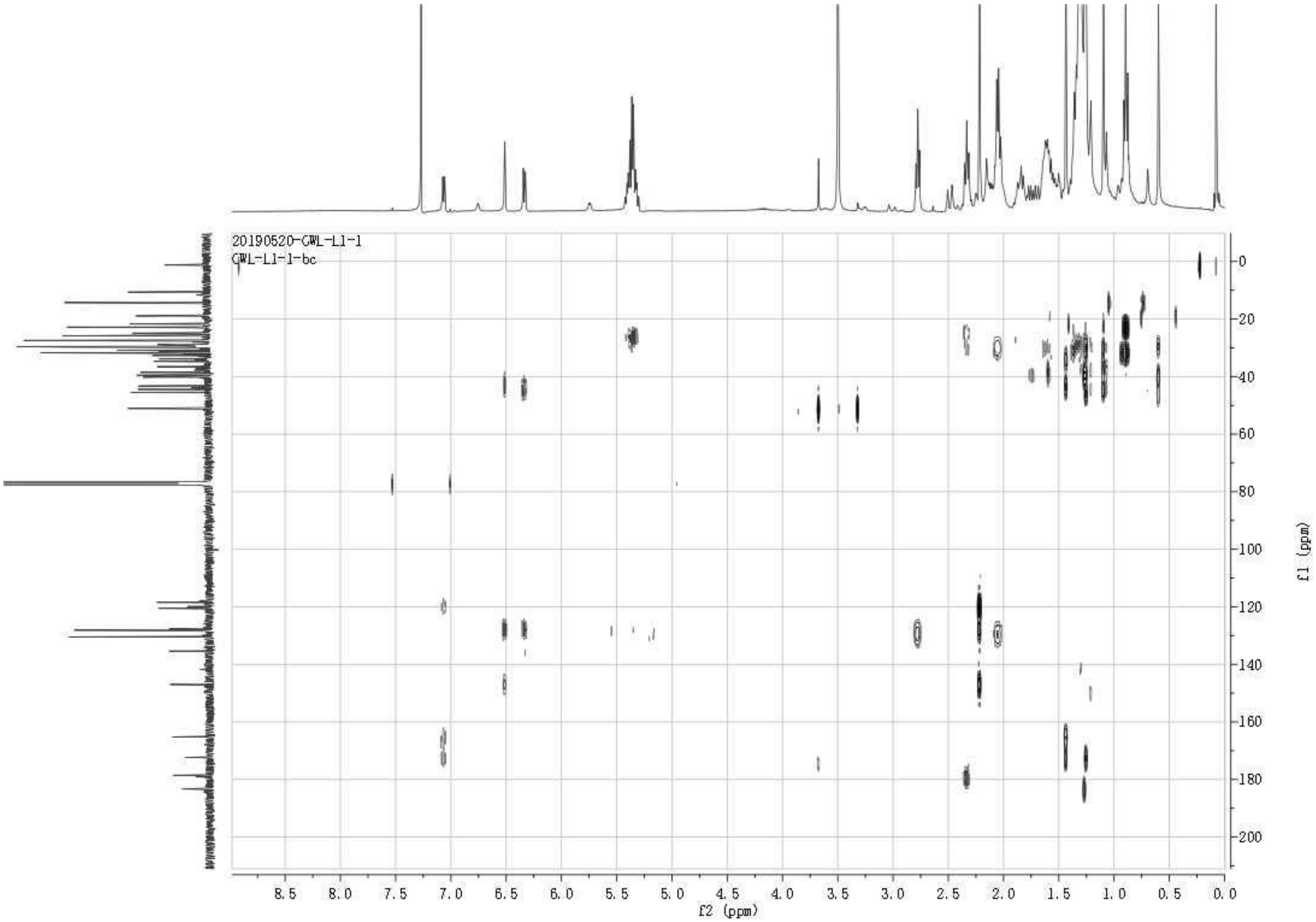

Figure S37 The HMBC spectrum of **4** in $CDCl_3$.

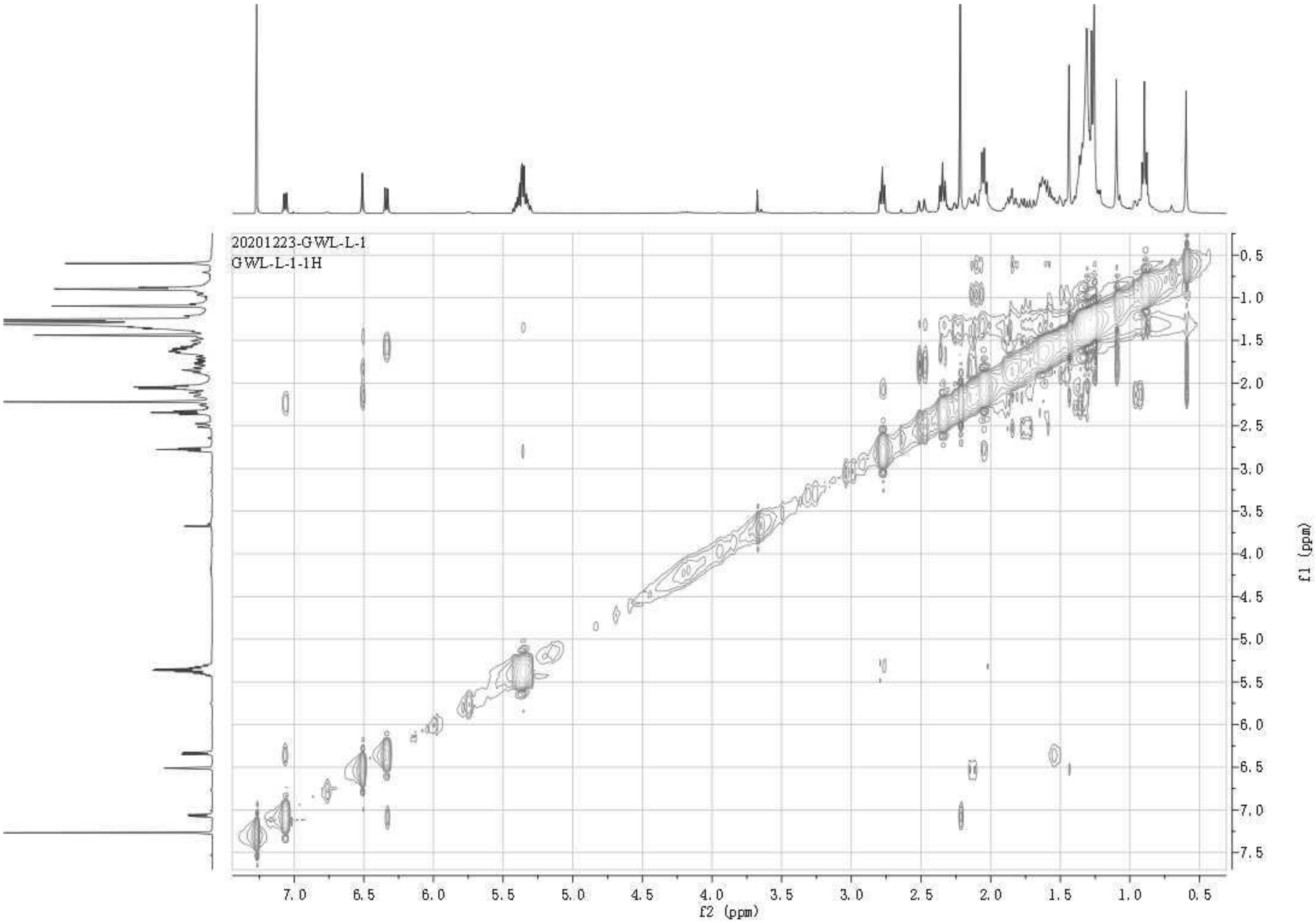

Figure S38 The NOESY spectrum of **4** in $CDCl_3$.

CCGTAGGGGTGAACCTGCGGAGGGATCATTGCTGGAACGCGCCCCAGGCGCACCCAGAAACCCTTTGTGAACTTATACCTTTTGTTGC
CTCGGCGCTGCTGGTCTTCACAGGCCCTTTGCTTCACAGCAAAGAGACGGCACGCCGGCGGCCAAGTTAACTATGTTTTTACACTGAA
ACTCTGAGAAAAAACACAAATGAATCAAAACTTTCAACAACGGATCTCTTGGTTCTGGCATCGATGAAGAACGCAGCGAAATGCGAT
AAGTAATGTGAATTGCAGAATTCAGTGAATCATCGAATCTTTGAACGCACATTGCGCCCTCTGGTATTCCGGAGGGCATGCCTGTTCG
AGCGTCATTTCAACCCTCAAGCATTGCTTGGTGTTGGGGCACTGCTTTTAACGAAGCAGGCCCTGAAATCTAGTGGCGAGCTCGCTAG
GACCCCGAGCGTAGTAGTTAAACCCTCGCTTTGGAAGGCCCTGGCGGTGCCCTGCCGTTAAACCCCCAACTTCTGAAAATTTGACCTC
GGATCAGGTAGGAATACCCGCTGAACTTAAGCATATCAATAAGCGGAGGAA

Figure S39 The ITS-18S sequencing of LGT-5 (578 bp in length).
